# Evolutionary rescue by aneuploidy in tumors exposed to anticancer drugs

**DOI:** 10.1093/genetics/iyaf098

**Published:** 2025-05-22

**Authors:** Remus Stana, Uri Ben-David, Daniel B Weissman, Yoav Ram

**Affiliations:** School of Zoology, Faculty of Life Sciences, Tel Aviv University, Tel Aviv 6997801, Israel; Department of Human Molecular Genetics and Biochemistry, Faculty of Medicine, Tel Aviv University, Tel Aviv 6997801, Israel; Department of Physics, Emory University, Atlanta, GA 30322, USA; School of Zoology, Faculty of Life Sciences, Tel Aviv University, Tel Aviv 6997801, Israel

**Keywords:** aneuploidy, evolutionary model, adaptive evolution, cancer, drug resistance, chromosome instability

## Abstract

Evolutionary rescue occurs when a population, facing a sudden environmental change that would otherwise lead to extinction, adapts through beneficial mutations, allowing it to recover and persist. A prime example of evolutionary rescue is the ability of cancer to survive exposure to treatment. One evolutionary mechanism by which a population of cancer cells can adapt to chemotherapy is aneuploidy. Aneuploid cancer cells can be more fit in an environment altered by anticancer drugs, in part because aneuploidy may disrupt the pathways targeted by the drugs. Indeed, aneuploidy is highly prevalent in tumors, and some anticancer drugs fight cancer by increasing chromosomal instability. Here, we model the impact of aneuploidy on the fate of a population of cancer cells. We use multitype branching processes to approximate the probability that a tumor survives drug treatment as a function of the initial tumor size, the rates at which aneuploidy and other beneficial mutations occur, and the growth rates of the drug-sensitive and drug-resistant cells. We also investigate the effect of the preexistent aneuploid cells on the probability of evolutionary rescue. Finally, we estimate the tumor’s mean recurrence time to revert to its initial size following treatment and evolutionary rescue. We propose that aneuploidy can play an essential role in the relapse of smaller secondary tumors.

## Teaser

Cancer cells can survive drug treatments through evolutionary rescue, where genetic changes enable persistence. Aneuploidy, an abnormal chromosome number, can aid drug resistance. Using models and simulations, Stana et al. examined its role in cancer survival. Aneuploidy delays extinction, increasing the chance of resistance mutations. The authors findings suggest therapies inducing aneuploidy may unintentionally promote resistance. Understanding these dynamics is crucial for designing better treatments to prevent relapse and improve cancer control.

## Introduction

### Aneuploidy in cancer

Each year, ∼10 million people die from cancer globally (Kocarnik *et al.* 2022). Understanding the factors that contribute to the failure of interventions is of great importance. One suggested factor is aneuploidy, in which cells are characterized by an imbalanced karyotype and alterations in the number of chromosomes (Schukken and Foijer 2018). Aneuploidy is caused by chromosomal instability and missegregation of chromosomes during mitosis. Changes in the number of chromosomes and chromosome arm copies allow cancer cells to survive under stressful conditions such as drug therapy (Rutledge *et al.* 2016; Ippolito *et al.* 2021; Lukow *et al.* 2021). Indeed, cancer cells are often aneuploid, and aneuploidy is associated with poor patient outcomes (Smith and Sheltzer 2018; Ben-David and Amon 2020).

Ippolito *et al.* (2021) induced aneuploidy in cancer cell lines by exposing them to reversine, a small-molecule inhibitor of the mitotic kinase Mpsi1, and then to anticancer drugs such as vemurafenib. Reversine-treated cells had a higher proliferation rate following drug exposure compared with sensitive cancer cells, due to selection of specific beneficial karyotypes. Similarly, Lukow *et al.* (2021) induced aneuploidy in cancer cells and observed that such cells have an advantage compared with sensitive cells during drug treatment despite having lower fitness before the onset of treatment. One proposed mechanism through which aneuploidy can confer resistance to anticancer drugs is by extending the length of the cell cycle, which prevents the drugs from damaging DNA and microtubules (Replogle *et al.* 2020). Other mechanisms include altering the expression of drug-metabolizing genes (Ippolito *et al.* 2021) and enhancing DNA repair due to chronically elevated baseline levels of DNA damage in aneuploid cells (Zerbib *et al.* 2024).

An essential aspect of aneuploidy is that the rate with which cells become aneuploid, that is, the rate of chromosome missegregation, is several orders of magnitude higher than the mutation rate (Bakker *et al.* 2023). A cell exposed to stress, such as chemotherapeutic drugs, can acquire aneuploidy faster than a mutation. Some proposed anticancer drugs elevate the missegregation rate to fight cancer cells (Lee *et al.* 2016), as an extremely high chromosome missegregation rate is incompatible with cell survival and proliferation.

### Evolutionary rescue

Populations adapted to a specific environment are vulnerable to environmental changes, which might cause the population’s extinction. Examples of such environmental changes include climate change, invasive species, and the onset of drug therapies. Adaptation is a race against time as the population size decreases in the new environment (Tanaka and Wahl 2022). *Evolutionary rescue* is the process by which the population acquires an adaptation that increases fitness in the new environment such that extinction is averted. There are three potential ways for a population to survive environmental change (Alexander *et al.* 2014; Bell 2017): migration to a new habitat similar to the one before the onset of environmental change (Harsch *et al.* 2014; Cobbold and Stana 2020; Zhou 2022); adaptation by phenotypic plasticity without genetic modification (Carja and Plotkin 2017, 2019; Gunnarsson *et al.* 2020; Levien *et al.* 2021); and adaptation through genetic modifications like mutation (Gomulkiewicz and Holt 1995; Uecker and Hermisson 2011, 2016; Orr and Unckless 2014; Uecker *et al.* 2014). Here, we focus on the latter.

There has been extensive theoretical analysis of evolutionary rescue. Orr and Unckless (2008, 2014) thoroughly investigated the simplest case in which there is a specific single mutation or low-frequency variant that is sufficient to rescue the population. Martin *et al.* (2013) extended these results to consider the effects of a range of rescue genotypes that can vary in both growth rate and variance in offspring number. Importantly for the present work, they also argued that two-step processes involving a “transient” intermediate genotype with growth rate close to zero may be an important form of rescue; however, they only calculated that the rate of such rescues should scale with the total number of such transient genotypes produced. Osmond *et al.* (2020) derived approximate expressions for the rate of these two-step rescues, allowing for a range of mutations, and found that for some parameter values two-step rescue is more likely than rescue by a single step.

Resistance to cancer therapy is a natural application of evolutionary rescue theory (Alexander *et al.* 2014). Iwasa *et al.* (2003) used multitype branching process theory to approximate the probability that a population under strong selective pressure can survive extinction, focusing on the case in which the cancer must get two specific mutations to be rescued, with the single-mutant still dying rapidly. Iwasa *et al.* (2004) used a similar approach to find simple approximate expressions for the probability that a single lineage would evolve to survive via a wide range of possible mutational pathways. These results are essentially the same as those described later in the general context by Osmond *et al.* (2020) for the probability of evolutionary rescue by multiple mutations, which we mentioned in the previous paragraph. A similar set of models describe adaptation to new environments (e.g. a pathogen evolving to cross a species barrier) and fitness valley crossing (Antia *et al.* 2003; Weissman *et al.* 2009). Gunnarsson *et al.* (2020) analyzed a model where a tumor consisting of two populations of cancer cells, one drug resistant and the other drug sensitive, can evade extinction by cells switching between the two phenotypes through epigenetic mutations. They found that even when drug-resistant cells are barely viable, the epimutations guarantee evolutionary rescue.

Here, we study evolutionary rescue after a sudden environmental change caused by initiating anticancer drug treatment. We consider a range of effects of aneuploidy, from tolerance to (partial) resistance to the drug (Brauner *et al.* 2016). Note that we use “tolerance” to refer to aneuploids that have negative growth rates, which is different from other references in the literature such as Berman and Krysan (2020). We estimate the effect of aneuploidy on the tumor’s evolutionary rescue probability. When aneuploidy provides drug resistance, it can directly rescue the tumor. However, when aneuploidy provides tolerance to the drug, it may act as an evolutionary “stepping stone” or “springboard” (Martin *et al.* 2013; Yona *et al.* 2015; Osmond *et al.* 2020), delaying extinction and thereby allowing the tumor more time to acquire a resistance mutation on top of the aneuploid background. As mentioned above, evidence suggests that aneuploidy may be a common strategy for tumor adaptation to drug therapy. Still, it is unknown how often aneuploidy provides tolerance and acts as a stepping stone. We also estimate the mean time until a tumor cell population reaches its pretreatment size following drug therapy. Given that aneuploidy is present in many tumors before the onset of therapy (Ben-David and Amon 2020; Lukow *et al.* 2021), we also consider the effect of pretreatment standing genetic variation on the evolutionary dynamics. Additionally, we are interested in the timescale of evolutionary rescue and the impact that aneuploidy has on the time necessary for the tumor to overcome drug therapy.

We address these questions by combining known results from the evolutionary rescue literature and considering the quantities and parameter values that are relevant for cancer. Because these results are scattered across multiple papers, and because they all follow from simple branching process approximations, we re-derive all results here for our model. While our focus is on the application of evolutionary rescue theory to the evolution of drug resistance in cancer, our model is a general one, and in the general evolutionary rescue context, this work can be seen as a characterization of the circumstances in which two-step processes contribute to rescue even when single-step rescue mutations are available, as in Osmond *et al.* (2020) and Iwasa *et al.* (2003, 2004).

## Models and methods

### Evolutionary model

We follow the number of cancer cells with one of three different genotypes at time *t*: sensitive, $s_{t}$; tolerant/resistant aneuploid, $a_{t}$; and resistant mutant, $m_{t}$. These cells divide and die with rates $\lambda_{k}$ and $\mu_{k}$ (for k=s,a,m). The division and death rate difference is r*_k_* = *λ*_*k*_−*μ_k_*. We assume the population of cells is under a strong stress, such as drug therapy, and therefore $r_{s}<0$, whereas the mutant is resistant to the stress, $r_{m}>0$. We consider a range of possible values for $r_{a}$, finding three distinct scenarios: in the first, aneuploid cells are partially resistant, $r_{m}>r_{a}>0$; in the second, aneuploid cells are tolerant, $0>r_{a}>r_{s}$; in the third, aneuploid cells are nongrowing, stationary, or growing or dying slowly, that is, either slightly tolerant or slightly resistant, such that $r_{a}\approx 0$, in a sense that we will make precise below. We define tolerant cells as those that survive drug exposure longer than sensitive cells, while resistant cells require higher drug concentrations to be affected. For details on these distinctions, see Brauner *et al.* (2016) .

We assume that both chromosomal missegregation and mutations occur during the process of mitosis. Sensitive cells may divide and then missegregate to become aneuploids at rate $u\lambda_{s}$. Both aneuploid and sensitive cells may divide and mutate to become mutants at rates $v\lambda_{a}$ and $v\lambda_{s}$, respectively. To model standing genetic variation, we assume that before the onset of therapy, sensitive cells become aneuploid with rate $\tilde{u}\lambda_{s}$ (which may differ from $u\lambda_{s}$) and that aneuploidy confers a fitness cost *c* in a drug-free environment, that is, we assume that aneuploid cells have an increased death rate compared with sensitive cells in a drug-free environment. We assume that *c* is not too small, so that standing variation is generated by a balance between the generation of aneuploids and selection against them, without the effect of genetic drift.

**Fig. 1.**
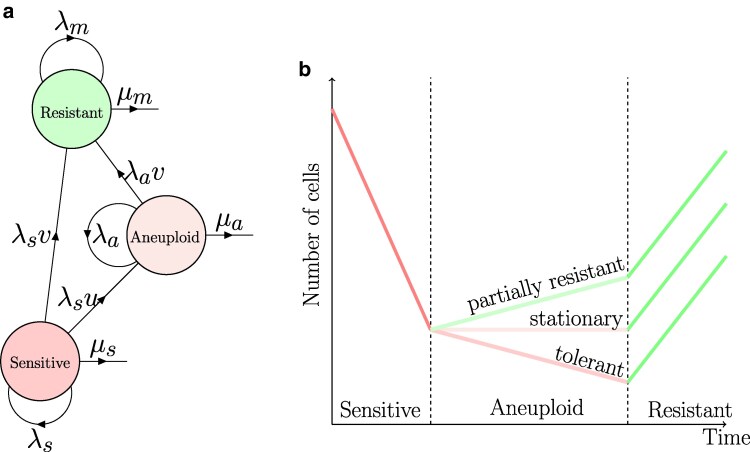
Model illustration. a) A population of cancer cells is composed of drug-sensitive, aneuploid, and mutant cells, which divide with rates $\lambda_{s}$, $\lambda_{a}$, and $\lambda_{m}$ and die at rates $\mu_{s}$, $\mu_{a}$, and $\mu_{m}$, respectively. Sensitive cells can divide and become aneuploid at rate $u\lambda_{s}$. Both aneuploid and sensitive cells can divide and acquire a mutation with rates $v\lambda_{a}$ and $v\lambda_{s}$, respectively. Color denotes the relative growth rates of the three genotypes such that $\lambda_{s}-\mu_{s}<\lambda_{a}-\mu_{a}<\lambda_{m}-\mu_{m}$. b) Sensitive cells are sensitive to the drug, while mutant cells are drug-resistant. The aneuploid may be tolerant, stationary, or partially resistant.

**Fig. 2.**
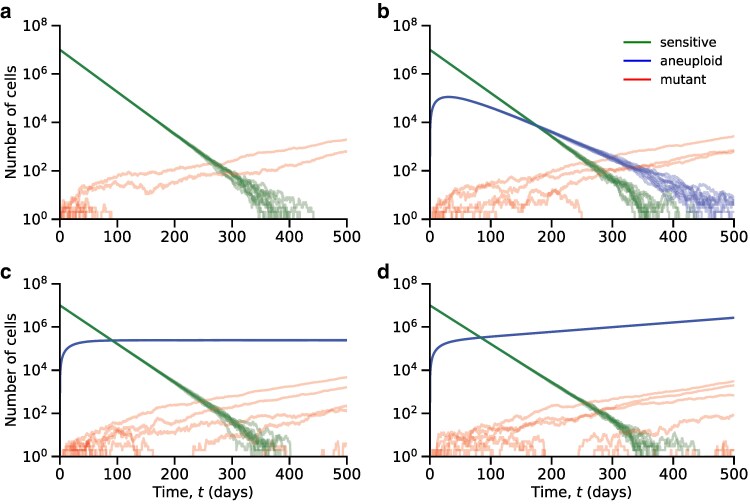
Sample trajectories of the genotype frequencies. a) Without aneuploidy (u=0), evolutionary rescue is possible through direct mutation, and in most scenarios, the tumor will become extinct due to the drug. b) When aneuploid cells are tolerant ($r_{a}<0$) evolutionary rescue can occur indirectly, but direct mutation is the most likely path for evolutionary rescue. c) When aneuploid cells are stationary ($r_{a}\approx 0$), we observe the appearance of mutant lineages even after the sensitive population has gone extinct, thus showing that stationary aneuploidy increases the probability of evolutionary rescue. d) When aneuploid cells are partially resistant ($r_{a}>0$), the tumor is rescued by the aneuploid cell population. Each plot shows 10 simulations of the number of sensitive, aneuploid, and mutant cells $\left(s_{t},a_{t},m_{t}\right)$ over time *t*. Here, $\lambda_{s}=0.1$, $\lambda_{m}=0.1$, $\mu_{s}=0.14$, $\mu_{a}=0.09$, $\mu_{m}=0.09$, $v=10^{-7}$, $N=10^{7}$; a) u=0; b) $\lambda_{a}=0.065$, $u=10^{-2}$; c) $\lambda_{a}=0.08999$, $u=10^{-2}$; d) $\lambda_{a}=0.095$, $u=10^{-2}$.

See Fig. 1 for a schematic representation of the model, Fig. 2 for sample trajectories of the different genotypes, and Table 1 for a summary of model parameters.

**Table 1.** Model parameters: Melanoma.

	Name	Value	Units	References
*N*	Initial tumor size	$10^{7}$ –$10^{9}$	cells	Del Monte (2009)
$\lambda_{s}$	Sensitive division rate	0.1	1/days	Bozic *et al.* (2013) and Rew and Wilson (2000)
$\mu_{s}$	Sensitive death rate	0.11 –0.17	1/days	Bozic *et al.* (2013)
$\lambda_{a}$	Aneuploid division rate^*a*^	0.06 –0.1	1/days	–
$\mu_{a}$	Aneuploid death rate^*a*^	0.09	1/days	–
$\lambda_{m}$	Mutant division rate	0.1	1/days	Bozic *et al.* (2013) and Rew and Wilson (2000)
$\mu_{m}$	Mutant death rate	0.09	1/days	Bozic *et al.* (2013) and Carlson (2003)
*u*	Missegregation rate	$10^{-2}$	1/cell division	Lee *et al.* (2016)
*v*	Mutation rate	$10^{-9}$ –$10^{-7}$	1/cell division	Bozic *et al.* (2013) and Loeb (2001)
$\tilde{u}$	Missegregation rate in the drug free environment^*a*^	$5\times 10^{-4}$ –$2\times 10^{-2}$	1/cell division	Shi and King (2005) and Thompson and Compton (2008)
*c*	Selection coefficient against aneuploidy in the drug free environment	0.07	1/days	Lukow *et al.* (2021)

Parameters from Bozic *et al.* (2013) consider patients with melanoma treated with the anticancer drug vemurfenib, in which resistance is conferred by trisomy in either Chr 2 or Chr 6. We have modified the parameters from Bozic *et al.* (2013) such that sensitive and mutant division rates are $\lambda_{s}=\lambda_{m}=\log\;\left(2\right)/T\approx 0.1$ instead of their value of 0.14, where *T* is the doubling time in the absence of cellular death obtained from Rew and Wilson (2000). For a discussion of the different interpretations of the tumor doubling times, see Avanzini and Antal (2019). Parameters marked with^*a*^ are not obtained from the literature.

## Stochastic simulations

Simulations are performed using the *Gillespie stochastic simulation algorithm* (Gillespie 1976, 1977) implemented in Python (Van Rossum and Drake 2009). The simulation monitors the number of cells of each type: sensitive, aneuploid, and mutant. Initially, the population starts with only sensitive cells, $s_{0}=N$, and the other genotypes are initially absent.

The cell population at time *t* is represented by the triplet $\left(s_{t},a_{t},m_{t}\right)$. The following describes the events that may occur (right column), the rates at which they occur (middle column), and the effect these events have on the population (left column, see Fig. 1):


$$\begin{array}{r} \begin{array}{ccc} (+1,0,0)\;: & \;\lambda_{s}s_{t}\left(1-u-v\right) & \left(\text{birth of sensitive cell}\right),\\ (-1,0,0)\;: & \;\mu_{s}s_{t} & \left(\text{death of sensitive cell}\right),\\ (0,+1,0)\;: & \;u\lambda_{s}s_{t} & (\text{sensitive cell divides}\\ & & \text{and becomes aneuploid}),\\ (0,0,+1)\;: & \;v\lambda_{s}s_{t} & (\text{sensitive cell divides}\\ & & \text{and becomes mutant}),\\ (0,+1,0)\;: & \;\lambda_{a}a_{t}\left(1-v\right) & \left(\text{birth of aneuploid cell}\right),\\ (0,-1,0)\;: & \;\mu_{a}a_{t} & \left(\text{death of aneuploid cell}\right),\\ (0,0,+1)\;: & \;v\lambda_{a}a_{t} & (\text{aneuploid cell divides}\\ & & \text{and becomes mutant}),\\ (0,0,+1)\;: & \;\lambda_{m}m_{t} & \left(\text{birth of mutant cell}\right),\\ (0,0,-1)\;: & \;\mu_{m}m_{t} & \left(\text{death of mutant cell}\right). \end{array} \end{array}$$

For the remainder of this paper, we assume that the division rates of sensitive and aneuploid cells can be written as $\lambda_{s}s_{t}(1-u-v)\approx \lambda_{s}s_{t}$ and $\lambda_{a}a_{t}(1-v)\approx \lambda_{a}a_{t}$ because u,v≪1 (Table 1). Each iteration of the simulation loop starts by computing the rates $\rho_{k}$ of each event *k*. We then draw the time until the next event, *δ*, from an exponential distribution whose rate parameter is the sum of the rates of all events, such that $\delta ∼\text{Exp}(\sum_{j}\rho_{j})$. Then, we randomly determine which event occurred, where the probability for event *k* is $p_{k}=\rho_{k}/\sum_{j}\rho_{j}$. Finally, we update the number of cells of each genotype according to the event that occurred and update the time from *t* to t+δ. We repeat these iterations until either the population becomes extinct (the number of cells of all genotypes is zero) or the number of mutant cells is high enough so that their extinction probability is <0.1%, that is, until


$$m_{t}>\left\lfloor \frac{3\log\;10}{\log\;\left(\lambda_{m}/\mu_{m}\right)}\right\rfloor +1,$$

which we obtain by solving $1-(1-p_{m}{)}^{m_{t}}=0.999$ for $m_{t}$ with $p_{m}=r_{m}/\lambda_{m}$ as the probability that a single mutant escapes stochastic extinction (Appendix A).

When simulations are slow (e.g. due to large population size) with runtimes in the order of days, we use *τ*-leaping (Gillespie 2001), where we assume that the change in the number of cells of genotype *k* in a fixed time interval *δ* is Poisson distributed with mean $\rho_{k}\delta$. If the number of cells of genotype *k* becomes negative, we change it to zero.

## Parameterization

We parametrize most of our simulations by considering melanoma cells and rely on Rew and Wilson (2000) and Bozic *et al.* (2013) for the division and death rates, respectively. Rew and Wilson (2000) report in vivo measurements of the potential doubling times (the waiting time for the number of cells in the tumor to double, disregarding cell death) for a large set of cancer types. The division rate is obtained as λ=log2/T≈0.1 per day. We take this to be the division rate for sensitive and assume that mutant cells, which are resistant to therapy, have the same division rate. Indeed, doubling times for tumors after relapse is lower than that of primary tumors for a variety of tissues (Tezuka *et al.* 2007; Rodgers *et al.* 2024) and metastatic melanoma has been shown to grow faster than primary melonoma (Carlson 2003).

Bozic *et al.* (2013) report the growth rate $r_{s}$ for sensitive melanoma cancer cells, from patient data, from which they deduce the death rate $0.11\leq \mu_{s}\leq 0.17$. We use $\mu_{s}=0.14$ per day. Additionally, they observed the growth rate of cancer cells before treatment to be 0.01, which we use as the growth rate of mutant cells, which are resistant to the drug. Thus, we use $\mu_{m}=0.1-0.01=0.09$ per day as the death rate for mutant cells.

The aneuploid death rate $\mu_{a}$ is set to the mutant death rate, $\mu_{m}=0.09$ per day, assuming aneuploidy increases resistance to the drug, such as cisplatin, by antagonizing cell division (Replogle *et al.* 2020). For the aneuploid growth rate to be intermediate between those of sensitive and mutant cells, $r_{s}\ll r_{a}\ll r_{m}$, we set the aneuploid division rate to be $0.06\leq \lambda_{a}\leq 0.1$. In most of our simulations, we use $\lambda_{a}=0.0899$ per day, so that aneuploid cells are tolerant and aneuploidy can only act as an evolutionary “stepping stone” for the generation of the resistant mutant that rescues the tumor (note that this mutant will occur on the background of an aneuploid genotype).

We also consider breast cancer cells, relying on Salehi *et al.* (2021), who studied multiple TNBC PDX clones (triple-negative breast cancer patient-derived xenografts). We chose three clones for which Salehi *et al.* (2021) report their relative Wrightian fitness, 1+s, in the presence of the drug cisplatin: TNBC-SA609 clone A with 1+s=1.047, TNBC-SA1035 clone H with 1+s=1.02, and TNBC-SA535 clone H with 1+s=1.01. The growth rate *Δ*, or the Malthusian fitness, is the log of the Wrightian fitness (Wu *et al.* 2013). We therefore have the following set of equations: $1.047=\exp\;\left(r_{SA609}-r_{s}\right),1.02=\exp\;\left(r_{SA1035}-r_{s}\right),1.01\;=\;\;$  $\exp\;\left(r_{SA535}-r_{s}\right)$. The growth rate of breast cancer cells in the absence of the drug is 0.0085 per day (Spratt *et al.* 1996). The doubling time of breast cancer cells in the absence of cellular death is 8.2 days (Rew and Wilson 2000). Thus, the division rate of breast cancer cells in the absence of the drug is log(2)/8.2=0.0845 per day, and their death rate is 0.076 per day. Salehi *et al.* (2021) report relative fitness for the aneuploid clones rather than division and growth rate. We, therefore, use the above estimates from breast cancer cells in the absence of drugs for the fittest clone, TNBC-SA609 clone A, and solve the set of equations assuming that the drug affects death rather than division and that mutant cells have the same division and death rates as the fittest aneuploid cells of TNBC-SA609 clone A. We therefore have $\lambda_{s}=\lambda_{a}=\lambda_{m}=0.0845$ for all clones, $\mu_{s}=0.1215$, $\mu_{SA609}=0.076$, $\mu_{SA1035}=0.1015$, $\mu_{SA535}=0.1115$, and $\mu_{m}=0.076$. These estimates are approximate at best (Table 2), and we hope that future experimental research could provide accurate estimates of these quantities.

The missegregation rate in cancer cells is estimated to be between $2.5\times 10^{-4}$ and $10^{-2}$ per chromosome per cell division (Shi and King 2005; Thompson and Compton 2008). Ippolito *et al.* (2021) observed that trisomy in Chr 2 and Chr 6 are most likely to confer increased resistance against the anticancer drug vemurafenib for A375 cells. We assume each of these trisomies is formed at the most likely rate, and as a result, we use $\tilde{u}=10^{-3}$ per cell division as the chromosome missegregation rate in the drug-free environment. Some drugs are known to increase chromosome instability (Mason *et al.* 2017 ; Wang *et al.* 2019 ). Specifically, Lee *et al.* (2016) estimated the effect of different anticancer drugs on the missegregation rate and found a 300% to 5,000% increase. We thus assume an anticancer drug that causes a 1,000% increase in the chromosome missegregation rate, which gives us $u=10^{-2}$ per cell division. We assume the mutation rate is $10^{-7}$ per gene per cell division (Loeb 2001), and since we assume that a single target gene confers resistance to the drug, we use $v=10^{-7}$ per cell division.

To estimate the fitness cost of aneuploidy, *c*, we note that Lukow *et al.* (2021) mixed sensitive and aneuploid A375 melanoma cells at 1:1 ratio, cultured them in a drug-free environment, and observed the ratio evolve as a function of time with the aneuploid cells declining to 15% after 24 days. Thus, our estimate for the fitness cost is c=|log(0.15/(1−0.15))/24|≈0.07 per day (Chevin 2011). We estimate the fraction of aneuploid cancer cells in the pretreatment environment using the formula $f=\tilde{u}\lambda_{s}/c$, which produces an estimate of $f=10^{-3}\times 10^{-1}/0.07$, that is, 0.14% of pretreatment cancer cells have the beneficial aneuploidy of interest.

Note that when we refer to drug-sensitive cells, we include those cells that have any aneuploidy that does not increase fitness in the presence of the drug (Table 1). Additionally, we focus on mutations that confer resistance, neglecting deleterious mutations (which are more common). We assume deleterious mutations and other aneuploidies occur at similar rates across genotypes and therefore neglect their effect on the dynamics.

## Density-dependent growth

In most of our analysis, we assume that cells from the initial population divide and die independently of each other. However, these cells will compete for resources. We assume this competition can be ignored because the drug will cause the cell density to rapidly drop below the carrying capacity where competition is important. To test this assumption, we simulate a logistic growth model, with division and death rates given by


$$\begin{array}{rl} \lambda_{s}^{\prime } & =\lambda_{s},\\ \mu_{s}^{\prime } & =\mu_{s}+\lambda_{s}\frac{s+a+m}{K},\\ \lambda_{a}^{\prime } & =\lambda_{a},\\ \mu_{a}^{\prime } & =\mu_{a}+\lambda_{a}\frac{s+a+m}{K},\\ \lambda_{m}^{\prime } & =\lambda_{m},\\ \mu_{m}^{\prime } & =\mu_{m}+\lambda_{m}\frac{s+a+m}{K}, \end{array}$$

where *K* is the tumor carrying capacity. The effective carrying capacity in this model is $K_{e}=Kr_{a}/\lambda_{a}\approx 10^{6}$ for $K=10^{8},\lambda_{a}=0.0901,$  $\mu_{a}=0.09$, where we define the effective carrying capacity to be the population size at which the aneuploid division rate is equal to the aneuploid death rate.

## Results

### Evolutionary rescue probability

In our model, *evolutionary rescue* occurs when drug-resistant cells appear and establish (avoid random extinction) in the population ($m_{t}\gg 1$) before the population becomes extinct ($s_{t}=a_{t}=m_{t}=0$). Aneuploidy may contribute to evolutionary rescue by either preventing (when $r_{a}>0$) or delaying (when $0>r_{a}>r_{s}$) the extinction of the population before mutant cells appear and establish. We assume independence between clonal lineages starting from an initial population of *N* sensitive cells (we check the effect of density-dependent growth on our results below) and therefore, we use multitype branching processes to model the dynamics of the cancer cell population. Multitype branching process models the growth and evolution of populations with distinct types, capturing dynamics like mutation and selection. In evolutionary rescue, it predicts the probability of population survival by tracking adaptive mutations that emerge and spread under environmental stress. Define $p_{s}$ as the probability that a lineage starting from a single drug-sensitive cell avoids extinction by acquiring drug resistance. Thus, $N^{*}=1/p_{s}$ is the threshold tumor size above which evolutionary rescue is likely (Iwasa *et al.* 2003), and the rescue probability is given by


(1) $$p_{\mathrm{rescue}}=1-{\left(1-p_{s}\right)}^{N}\approx 1-e^{-Np_{s}}=1-e^{-N/N^{*}},$$

where the approximation $(1-p_{s})\approx e^{-p_{s}}$ assumes that $p_{s}$ (but not necessarily $Np_{s}$) is small. Indeed, when $N<1/p_{s}$, then the probability for evolutionary rescue is $p_{\mathrm{rescue}}\approx Np_{s}$, and when $N>1/p_{s}$, it is $p_{\mathrm{rescue}}\approx 1$, justifying the definition of $N^{*}$ as the threshold tumor size for evolutionary rescue.

We use multitype branching-process theory to find approximate expressions equations (A5), (A8), and (A12) for $p_{s}$ in three distinct scenarios (Appendix A). Substituting these into $N^{*}=1/p_{s}$, we find approximations for the threshold tumor size, $N^{*}$. In these approximations, an important quantity is $T^{*}=\sqrt{\lambda_{m}/4v\lambda_{a}^{2}r_{m}}$, which is the critical time an aneuploid lineage needs to survive to produce a resistant mutant that avoids random extinction. First, if aneuploidy is sufficiently rare ($u\lambda_{a}T^{*}<1$), or if aneuploidy is rare ($u\lambda_{a}<-r_{a}$) and sensitive to the drug ($r_{a}T^{*}<-1$), then it is likely that evolutionary rescue will occur through a direct resistance mutation in a sensitive cell without aneuploidy playing a role in the adaptive dynamics, such that


(2) $$N_{m}^{*}\approx \frac{\left|r_{s}\right|}{v\lambda_{s}}\frac{\lambda_{m}}{r_{m}},$$

which is similar to a classical result by Orr and Unckless (2008). Here, $\left|r_{s}|/(v\lambda_{s}\right)$ is the ratio of the rate at which sensitive cells decrease in number and the rate at which they are mutating. Notably, the aneuploidy parameters (*u*, $\lambda_{a}$, $\mu_{a}$) do not affect $N_{m}^{*}$.

Otherwise, aneuploidy is frequent enough ($u\lambda_{a}>\max$  $\left(-r_{a},1/T^{*}\right)$) to affect the evolution of drug resistance. The threshold tumor size can be approximated by one of the following scenarios, depending on $r_{a}T^{*}$, which represents the change in the aneuploid log-population size during the critical time,


(3) $$N_{a}^{*}\approx \frac{\left|r_{s}\right|}{u\lambda_{s}}\cdot \left\{ \begin{array}{cc} \frac{\left|r_{a}\right|}{v\lambda_{a}}\frac{\lambda_{m}}{r_{m}},\; & r_{a}T^{*}\ll -1\\ & (\text{tolerant aneuploids}),\\ 2\lambda_{a}T^{*}, & -1\ll r_{a}T^{*}\ll 1\\ & (\text{stationary aneuploids}),\\ \frac{\lambda_{a}}{r_{a}},\; & r_{a}T^{*}\gg 1\\ & (\text{resistant aneuploids}). \end{array}\right.$$

**Fig. 3.**
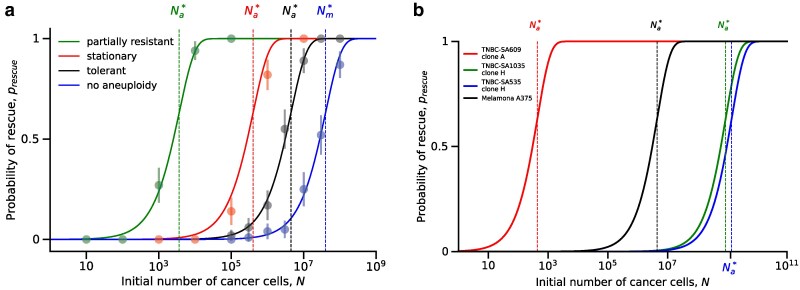
Aneuploidy facilitates the evolutionary rescue of tumors under drug treatment. The probability of evolutionary rescue, $p_{\mathrm{rescue}}$, as a function of the initial tumor size, *N* (equation (1)). Dashed vertical lines show the threshold tumor size, $N_{a}^{*}$, above which the probability is high (equation (3)). a) Blue dashed line: without aneuploidy (u=0). Black line: tolerant aneuploidy ($u=10^{-2},\lambda_{a}=0.0899$). Red line: stationary aneuploidy ($u=10^{-2},\lambda_{a}=0.08999$). Green line: partially resistant aneuploidy ($u=10^{-2},\lambda_{a}=0.095$). Markers and error bars for averages of simulation results with 95% confidence interval ($p\pm 1.96\sqrt{\;p(1-p)/n}$, where *p* is the fraction of simulations in which the tumor has been rescued, and n=100 is the number of simulations). Parameters: $\lambda_{s}=0.1,\lambda_{m}=0.1,\mu_{s}=0.14,\mu_{a}=0.09,\mu_{m}=0.09,v=10^{-7}$. b) Comparison for melanoma and breast cancer with parameters from the literature (Tables 1 and 2). Black line: Melanoma A375. Blue line: Breast cancer TNBC-SA1035 clone H. Red line: Breast cancer TNBC-SA609 clone A. Green line: Breast cancer TNBC-SA535 clone H.

**Fig. 4.**
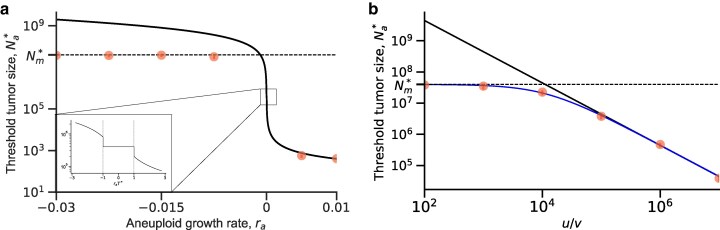
Aneuploidy reduces tumor threshold size. a) The threshold tumor size $N_{a}^{*}$ (equation (3)) as a function of the aneuploid growth rate $r_{a}$. The dashed horizontal line shows $N_{m}^{*}$ (equation (2)), the threshold tumor size without aneuploidy (u=0). When aneuploid growth rate is close to or higher than zero, aneuploidy decreases the threshold tumor size, facilitating evolutionary rescue. The inset highlights the scenario when aneuploid cells are stationary. Red dots for simulations and error bars for the 95% confidence intervals obtained with bootstrap (Appendix G). Parameters: $\lambda_{s}=0.1,\lambda_{m}=0.1,\mu_{s}=0.14,\mu_{a}=0.09,\mu_{m}=0.09,u=10^{-2},v=10^{-7}$. b) Threshold tumor size $N_{a}^{*}$ (equation (3)) as a function of the ratio of aneuploidy and mutation rates, u/v. Dashed horizontal line shows $N_{m}^{*}$ (equation (2)), the threshold tumor size without aneuploidy (u=0). When the aneuploidy rate is much higher than the mutation rate, aneuploidy decreases the threshold tumor size, facilitating evolutionary rescue. Blue line represents the exact formula for threshold tumor size $N_{a}^{*}$ while the solid black line represents the approximation (equation (3)). Red dots represent simulation results, and the error bars represent the 95% confidence intervals obtained with bootstrap (Appendix G). Parameters: $\lambda_{s}=0.1,\lambda_{a}=0.0899,\lambda_{m}=0.1,\mu_{s}=0.14,\mu_{a}=0.09,\mu_{m}=0.09,v=10^{-7}$.

This equation is equivalent to equation A3 of Iwasa *et al.* (2004) and to equation 8 of Osmond *et al.* (2020) with the distribution of fitness effects set to a delta function; our “tolerant,” “stationary,” and “resistant” scenarios correspond to Osmond *et al.* (2020)’s “sufficiently subcritical,” “sufficiently critical,” and “sufficiently supercritical,” respectively. These approximations are accurate when compared with results of stochastic evolutionary simulations (Figs. 3 and 4). Our parameterization for triple-negative breast cancer clones suggests that TNBC-SA609 clone A is resistant to the drug ($r_{a}T^{*}>500$), whereas TNBC-SA1035 clone H and TNBC-SA535 clone H are tolerant ($r_{a}T^{*}<-1000$).

**Table 2.** Model parameters: Breast cancer.

	Name	Value	Units
$\lambda_{s}$	Sensitive division rate	0.0845	1/days
$\mu_{s}$	Sensitive death rate	0.1215	1/days
$\lambda_{a}$	Aneuploid division rate	0.0845	1/days
$\mu_{a}$	Aneuploid death rate	0.076 , 0.1015, 0.1115	1/days
$\lambda_{m}$	Mutant division rate	0.0845	1/days
$\mu_{m}$	Mutant death rate	0.076	1/days

The derivation of these values, described in the methods section, uses fitness estimates from Salehi *et al.* (2021). Parameters not listed here remain unchanged from the values provided in Table 1.

In the first scenario, the treatment effectively kills aneuploid cells but not as quickly as it kills sensitive cells. In the second scenario, aneuploid cells are sufficiently resistant, and the expected size of each aneuploid lineage is roughly one. In both of these scenarios, aneuploidy increases the probability of rescue by slowing or halting the decrease in the tumor population size, allowing more opportunities to produce resistant mutants. In the third scenario, aneuploid cells are sufficiently resistant for the population to re-grow the tumor without additional resistance mutations. Notably, in this scenario the mutant parameters (*v*, $\lambda_{m}$, and $r_{m}$) do not affect $N_{a}^{*}$ beyond their effect on $T^{*}$. In all scenarios, $N_{a}^{*}$ is proportional to 1/u such that increasing the missegregation rate *u* will decrease the threshold tumor size (Fig. 4b). Furthermore, increasing the aneuploid growth rate $r_{a}$ (which appears both in the terms and in the conditions), also reduces the threshold tumor size, with a sharp decrease around $r_{a}=0$, but the effect is minor when $\left|r_{a}\right|$ is small compared with $T^{*}$ as this would result in the second scenario where $dN_{a}^{*}/dr_{a}=0$ (Fig. 4a). The tumor threshold size decreases with the mutation rate in the first and second scenarios: $N_{a}^{*}$ is proportional to 1/v in the first scenario (tolerant aneuploids) and to $\sqrt{1/v}$ in the second scenario (stationary aneuploids). Furthermore, the growth rate $r_{a}<0$ that allows tolerant aneuploids to rescue the tumor is between $-u\lambda_{a}$ and $-1/T^{*}$, which is proportional to $-\sqrt{v}$. Thus, increasing the mutation rate *v* will decrease the tumor threshold size $N_{a}^{*}$, making evolutionary rescue more likely.

Using equations (2) and (3), we can find the ratio of threshold tumor size for rescue via aneuploidy (*u* is high) or via direct mutation (*u* is low),


(4) $$\frac{N_{a}^{*}}{N_{m}^{*}}\approx \left\{ \begin{array}{cc} \frac{\left|r_{a}\right|}{u\lambda_{a}},\; & r_{a}T^{*}\ll -1\\ & (\text{tolerant aneuploids}),\\ \frac{1}{u}{\left(v\frac{r_{m}}{\lambda_{m}}\right)}^{1/2},\; & -1\ll r_{a}T^{*}\ll 1\\ & (\text{stationary aneuploids}),\\ v\frac{r_{m}}{\lambda_{m}}{\left(u\frac{r_{a}}{\lambda_{a}}\right)}^{-1},\; & r_{a}T^{*}\gg 1\\ & (\text{resistant aneuploids}). \end{array}\right.$$

Importantly, when this threshold is smaller than 1, aneuploidy is expected to play a role in evolutionary rescue, as it reduces the threshold tumor size needed for rescue. As expected, this ratio increases with the mutation rate *v* and decreases with the aneuploidy rate *u*. In the first scenario, $|r_{a}|/u\lambda_{a}$ is the ratio of the number of sensitive divisions required to produce an aneuploid, 1/u, to the number of divisions in the resulting aneuploid lineage before it goes extinct, $\lambda_{a}/|r_{a}|$. In the third scenario, $\left(v\frac{r_{m}}{\lambda_{m}})/(u\frac{r_{a}}{\lambda_{a}}\right)$ is the ratio of the rates of appearance of resistant mutants that avoid extinction and partially resistant aneuploids that avoid extinction. In the second scenario, $\frac{1}{u}(v\frac{r_{m}}{\lambda_{m}}{)}^{1/2}=\sqrt{\frac{r_{a}}{u\lambda_{a}}v\frac{r_{m}}{\lambda_{m}}(u\frac{r_{a}}{\lambda_{a}}{)}^{-1}}$, which is the geometric mean of the first and third scenarios.

Interestingly, increasing both the aneuploid division rate, $\lambda_{a}$, and the aneuploid death rate, $\mu_{a}$, such that the growth rate $r_{a}$ remains constant, leads to a decrease in $T^{*}$, pushing the system to the second scenario. This is because increasing $\lambda_{a}$ causes a decrease in $T^{*}$ as it increases the effective mutation rate $v\lambda_{a}$ (as mutations mostly occur during division) and a lineage does not have to survive as long in order to generate a successful mutant. In the second scenario, the threshold tumor size $N_{a}^{*}$ is unaffected by the division rate $\lambda_{a}$ (i.e. $d\lambda_{a}T^{*}/d\lambda_{a}=0$). Thus, if aneuploid cells rapidly die due to the drug but compensate by rapidly dividing, increasing the division rate will *not* facilitate adaptation.

We can categorize tumors by their size: small tumors with size $N<N_{a}^{*}$ that are unlikely to survive treatment, intermediate tumors with size $N_{a}^{*}<N<N_{m}^{*}$ that rely on aneuploidy for evolutionary rescue, and large tumors with size $N>N_{m}^{*}$ that could overcome drug treatment without aneuploidy (Supplementary Fig. 3). For the parameter values in Table 1 with $\lambda_{a}=0.0899,\mu_{s}=0.14,$  $u=10^{-2},v=10^{-7}$, we are in the tolerant aneuploid scenario, and substituting in equations (2) and (3), we have $N_{a}^{*}\approx 4\times 10^{6}$ and $N_{m}^{*}\approx 4\times 10^{7}$. Hence, we obtain the ratio $N_{a}^{*}/N_{m}^{*}\approx 0.11$ (equation (4)), that is, aneuploidy reduces the threshold tumor size by 89%. Interestingly, the threshold between small and intermediate tumors, $N_{a}^{*}$, is similar to the tumor detection threshold of $4.19\times 10^{6}$ cells for a wide variety of tumors (Avanzini and Antal 2019). We note that vemurafenib-treated melanomas (i.e. melanomas with sizes above the detection threshold) have a probability >50% to relapse (Handa *et al.* 2022; Piejko *et al.* 2023).

Aneuploidy may lead to an increased mutation rate in cancer cells (Janssen *et al.* 2011; Passerini *et al.* 2016; Garribba *et al.* 2023). Thus, we extended our model to account for this in Appendix H. We find that increasing the mutation rate in aneuploid cells by one order of magnitude leads to a decrease in the threshold tumor size of approximately one order of magnitude (Supplementary Fig. 10). Also, it transitions the system from the first scenario (tolerant aneuploids) to the second scenario (stationary aneuploids) without changing the aneuploid growth rate, $r_{a}$.

In our analysis, we used branching processes, which assume that growth (division and death) is density-independent. However, growth may be limited by resources (oxygen, nutrients, etc.) and therefore depend on cell density. We performed stochastic simulations of a logistic growth model with a carrying capacity. We find that our density-independent approximations agree with the results of simulations with density-dependent growth for biologically relevant parameter values (Supplementary Fig. 1).

#### Standing vs. de novo genetic variation

We initially assumed that at the onset of drug treatment, the tumor consisted entirely of drug-sensitive cells. In reality, aneuploidy is likely generated even before treatment begins, at some rate $\tilde{u}$, which may be lower in the absence of drugs, $\tilde{u}<u$ (Mason *et al.* 2017; Wang *et al.* 2019). Aneuploidy is also thought to carry a fitness cost *c* when drugs are absent (Giam and Rancati 2015; Replogle *et al.* 2020). Therefore, if the tumor size *N* is large—as expected at the time of treatment—a fraction of cells, approximately $f\approx \tilde{u}\lambda_{s}/c$, may already be aneuploid. Here we assume that the drug affects the sensitive death rate but not the division rate and therefore we use $\lambda_{s}$ for the sensitive division rate in the drug-free environment.

Therefore, the threshold tumor size for rescue by standing genetic variation, ${\tilde{N}}_{a}^{*}$, is similar to the threshold for rescue by de novo variation, $N_{a}^{*}$, except that the sensitive growth rate $\left|r_{s}\right|$ is replaced by the cost of aneuploidy *c*, such that


(5) $$\frac{{\tilde{N}}_{a}^{*}}{N_{a}^{*}}=\frac{u}{\tilde{u}}\frac{c}{\left|r_{s}\right|}.$$

**Fig. 5.**
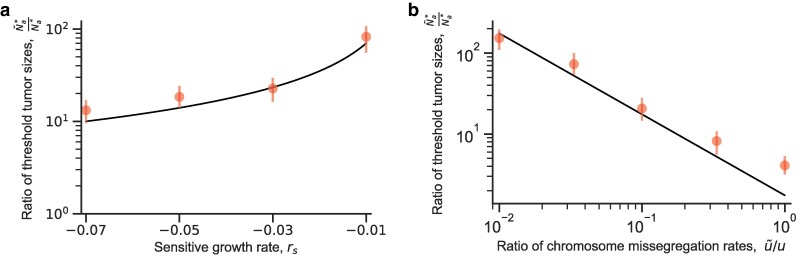
Standing genetic variation facilitates the evolutionary rescue of tumors under drug treatment. a) Ratio of threshold tumor sizes for rescue by standing genetic variation and by de novo variation, ${\tilde{N}}_{a}^{*}/N_{a}^{*}$, when a fraction $\frac{\tilde{u}\lambda_{s}}{c}$ is aneuploid at the start of treatment, as a function of the sensitive growth rate $r_{s}$. Standing genetic variation will drive drug resistance when the sensitive cell population is rapidly declining ($r_{s}\ll 0$) due to a stronger effect of the drug on sensitive cells. Red dots represent simulation results, and the error bars represent the 95% confidence intervals obtained with bootstrap (Appendix G). Parameters: $\lambda_{s}=0.1,\lambda_{a}=0.0899,\lambda_{m}=0.1,\mu_{a}=0.09,\mu_{m}=0.09,\tilde{u}=10^{-3},u=10^{-2},v=10^{-7}$. b) Ratio of threshold tumor size ${\tilde{N}}_{a}^{*}$ (when a fraction $\frac{\tilde{u}\lambda_{s}}{c}$ is aneuploid at the start of treatment) and $N_{a}^{*}$ as a function of the ratio of aneuploidy rates $\tilde{u}/u$. De novo aneuploids will have a larger contribution to the appearance of drug resistance if the drug induces the appearance of aneuploid cells ($u\gg \tilde{u}$). Red dots represent simulation results, and the error bars represent the 95% confidence intervals obtained with bootstrap (Appendix G). Parameters: $\lambda_{s}=0.1,\lambda_{a}=0.0899,\lambda_{m}=0.1,\mu_{s}=0.14,\mu_{a}=0.09,\mu_{m}=0.09,\tilde{u}=10^{-3},v=10^{-7}$.

This result has been previously reported by Orr and Unckless (2008) and Martin *et al.* (2013) for one-step evolutionary rescue. Comparing this approximation of ${\tilde{N}}_{a}^{*}/N_{a}^{*}$ to results of stochastic simulations, we find that the approximations are accurate (Fig. 5). Standing genetic variation will drive evolutionary rescue if sensitive cells die rapidly (growth rate $r_{s}$ is negative) due to a strong effect of the drug on sensitive cells or if the cost of aneuploidy in the drug-free environment, *c*, is small. In contrast, de novo aneuploid cells will have a greater contribution to rescue if the cost of aneuploidy, *c*, is large, the effect of the drug on sensitive cells is weak ($r_{s}$ is close to zero), or if the drug induces the appearance of aneuploid cells ($u>\tilde{u}$). For example, with $\lambda_{s}=0.1$, $\mu_{s}=0.14$, $u=10^{-2}$, $\tilde{u}=10^{-3}$, and c=0.07, the ratio of the threshold tumor sizes for standing vs. de novo variation is ${\tilde{N}}_{a}^{*}/N_{a}^{*}\approx 17.5$, which means that de novo genetic variation is the main driver of evolutionary rescue.

Using equation (2), (3), and (5), we can find the ratio of threshold tumor size for rescue via standing genetic variation to the threshold for rescue via mutation,


(6) $$\begin{array}{rl} \frac{{\tilde{N}}_{a}^{*}}{N_{m}^{*}} & =\frac{{\tilde{N}}_{a}^{*}}{N_{a}^{*}}\frac{N_{a}^{*}}{N_{m}^{*}}\\ & \approx \frac{c}{\left|r_{s}\right|}\left\{ \begin{array}{cc} \frac{\left|r_{a}\right|}{\tilde{u}\lambda_{a}},\; & r_{a}T^{*}\ll -1\\ & (\text{tolerant aneuploids}),\\ \frac{1}{\tilde{u}}{\left(v\frac{r_{m}}{\lambda_{m}}\right)}^{1/2},\; & -1\ll r_{a}T^{*}\ll 1\\ & (\text{stationary aneuploids}),\\ v\frac{r_{m}}{\lambda_{m}}{\left(\tilde{u}\frac{r_{a}}{\lambda_{a}}\right)}^{-1},\; & r_{a}T^{*}\gg 1\\ & (\text{resistant aneuploids}). \end{array}\right. \end{array}$$

Evolutionary rescue through direct mutation is more likely if the cost of aneuploidy, *c*, is large or the effect of the drug, $r_{s}$, is small. In contrast, standing genetic variation will drive adaptation if the pretreatment chromosome missegregation rate, $\tilde{u}$, is large. The ratio does not depend on the rate of chromosome missegregation induced by the drug, *u*. However, if the aneuploid growth rate, $r_{a}$, increases, evolutionary rescue is driven by standing genetic variation. ‘ of $\lambda_{s}=0.1$, $\lambda_{a}=0.0899$, $\lambda_{m}=0.1$, $\mu_{s}=0.14$, $\mu_{a}=0.09$, $\mu_{m}=0.09$, $\tilde{u}=10^{-3}$, and $v=10^{-7}$, we are in the first scenario (tolerant aneuploids) and obtain the ratio ${\tilde{N}}_{a}^{*}/N_{m}^{*}\approx 1.94$, which means that standing genetic variation does not drive evolution of drug resistance when compared with direct mutation. We note that for larger values of the pretreatment chromosome missegregation rate, $\tilde{u}$, which are consistent with empirical studies (Table 1), standing genetic variation can drive adaptation when compared with direct mutation.

## Recurrence time due to evolutionary rescue

When evolutionary rescue occurs, the time until the tumor recurs may still be long. We therefore explored the time until the tumor recurs, that is, the time until the tumor reaches its original size, *N*. When the expected number of resistant lineages that avoid extinction is small, the expected recurrence time can be estimated by adding two terms: the *mean evolutionary rescue time*, which is the waiting time for the appearance of a resistant lineage that avoids extinction (conditioned on such an event occurring in the first place), and the *mean proliferation time*, which is the expected time for that lineage to grow to *N* cells. However, when the expected number of resistant lineages is large, the mean recurrence time cannot be separated into the mean evolutionary rescue time and mean proliferation time because multiple mutant lineages contribute towards the mutant population size reaching the initial tumor size. In this case the dynamics of the number of mutant cells is deterministic and can therefore be modeled by a system of ordinary differential equations (ODEs), which describe how the number of mutant cells changes over time by its time derivative (equation (D2)). Of particular interest is the distribution of the evolutionary rescue time and recurrence time with tolerant aneuploid cells ($r_{a}T^{*}\ll 1$) as it represents the two-step evolutionary rescue scenario. We focus on the parameter values in Table 1 with $\lambda_{a}=0.0899$ (tolerant aneuploids), $\mu_{s}=0.14$ (intermediate sensitive death rate), and $v=10^{-7}$ (high end of the mutation rate).

### Evolutionary rescue time

We have derived approximations for $\tau_{m}$, the mean evolutionary rescue time without aneuploidy (u=0), and $\tau_{a}$, the mean rescue time with aneuploidy (u>0), both conditioned on evolutionary rescue occurring (Appendix C). These approximations agree with simulation results for small, intermediate, and large tumor sizes (Supplementary Figs. 2 and 6 ). The mean rescue time with aneuploidy for small and large tumors follows:


(7) $$\tau_{a}\approx \left\{ \begin{array}{cc} -\frac{1}{r_{s}}-\frac{1}{r_{a}},\; & N\ll N_{a}^{*},\\ \frac{1}{v\lambda_{s}N}\frac{\lambda_{m}}{r_{m}},\; & N\gg N_{m}^{*}. \end{array}\right.$$

For small tumors ($N\ll N_{a}^{*}$), the mean rescue time is the two-step equivalent of the one-step result from Orr and Unckless (2014, expectation of equation 18). The mean rescue time (conditioned on rescue occurring) is an increasing function of the sensitive and aneuploid growth rates and independent of the other model parameters, including tumor size (blue line in Supplementary Fig. 6). This is because if the population rapidly declines but is then rescued, than the resistance mutation must have appeared early; if the population slowly declines, than mutations can appear later and the mean time will be longer. In our focus parameter regime, we have $r_{s}=-0.04$ and $r_{a}=-10^{-4}$, such that the mean rescue time is mainly determined by the aneuploid growth rate, and $\tau_{a}\approx 10^{4}$ days (equation (7)).

For large tumors ($N\gg N_{m}^{*}$), the dynamics are equivalent to a scenario where rescue mutations appear at a constant rate, and the mean rescue time is independent of the aneuploid parameters (*u*, $\lambda_{a}$, and $r_{a}$). Increasing the per division mutation rate, *v*, leads to the faster appearance of a rescue mutations and hence reduced mean rescue time. Increasing the tumor size leads to shorter mean rescue time, as more sensitive cells can mutate to become resistant.

Given that a fraction f≈0.14% of the initial cancer cell population is expected to have beneficial aneuploidy even before the onset of drug treatment, we want to know whether the mean evolutionary rescue time is affected by the standing genetic variation. We calculated the mean evolutionary rescue time with standing genetic variation, ${\tilde{\tau }}_{a}$ (equation (C10)), and compared our result with simulations (Supplementary Fig. 9). We find that standing genetic variation does not significantly affect the mean evolutionary rescue time.

**Fig. 6.**
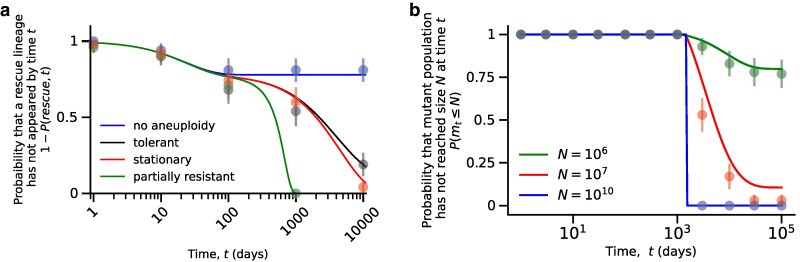
Aneuploidy extends the window of opportunity for evolutionary rescue of tumors under drug treatment. a) The probability that a successful mutant has not appeared by time *t*. Black line: tolerant aneuploidy ($u>0,\lambda_{a}=0.0899$). Red line: stationary aneuploidy ($u>0,\lambda_{a}=0.089999$). Green line: partially resistant aneuploidy ($u>0,\lambda_{a}=0.095$). Blue line: no aneuploidy (u=0). Aneuploidy plays an important role in rescuing the tumor cell population as the sensitive population becomes extinct. Markers represent simulation results, and the error bars represent 95% confidence interval ($p\pm 1.96\sqrt{\;p(1-p)/n}$ where *p* is the fraction of simulations in which a successful mutant has not been generated, and n=100 is the number of simulations). Parameters: $\lambda_{s}=0.1,\lambda_{m}=0.1,\mu_{s}=0.14,\mu_{a}=0.09,\mu_{m}=0.09,u=10^{-2},v=10^{-7},N=10^{7}$. b) Probability that a mutant cancer cell population has not reached size *N* at time *t* when aneuploidy provides tolerance. Green line: $N=10^{6}$ (small tumor). Red line: $N=10^{7}$ (intermediate-sized tumor). Blue line: $N=10^{10}$ (large tumor). Increasing the initial tumor size guarantees that the cancer will relapse. Markers represent simulations, and the error bars represent 95% confidence interval ($p\pm 1.96\sqrt{\;p(1-p)/n}$ where *p* is the fraction of the simulations in which the mutant population size has not reached *N* and n=100 is the number of simulations). Parameters: $\lambda_{s}=0.1,\lambda_{a}=0.0899,\lambda_{m}=0.1,\mu_{s}=0.14,\mu_{a}=0.09,\mu_{m}=0.09,u=10^{-2},v=10^{-7}$.

We calculate the probability that a rescue mutation has been generated by time *t* in Appendix E. This allows us to examine whether aneuploidy promotes or delays evolutionary rescue. We find that aneuploidy promotes evolutionary rescue after $1/r_{s}\approx 100$ days, at a time when no more rescue mutations are generated through mutations in sensitive cells (Fig. 6a). Thus, aneuploidy increases the *window of opportunity* for evolutionary rescue. This can have a counter-intuitive outcome: conditioned on the rescue of the tumor, tumors rescued by aneuploid cells may acquire rescue mutations later than those rescued by sensitive cells.

### Recurrence time

We next approximated the mean time for the population of mutant cancer cells to reach the initial, pretreatment population size *N*, which we denote the recurrence time $\tau_{a}^{r}$ (equations (7), (D1), and (D4)),


(8) $$\tau_{a}^{r}\approx \left\{ \begin{array}{cc} -\frac{1}{r_{s}}-\frac{1}{r_{a}}+\frac{\log\;p_{m}N}{r_{m}},\; & N\ll N_{a}^{*},\\ \frac{1}{r_{m}}\log\;\frac{r_{m}-r_{s}}{v\lambda_{s}},\; & N\gg N_{m}^{*}, \end{array}\right.$$

where $p_{m}$ is the probability that a lineage starting from a single mutant cell escapes stochastic extinction.

**Fig. 7.**
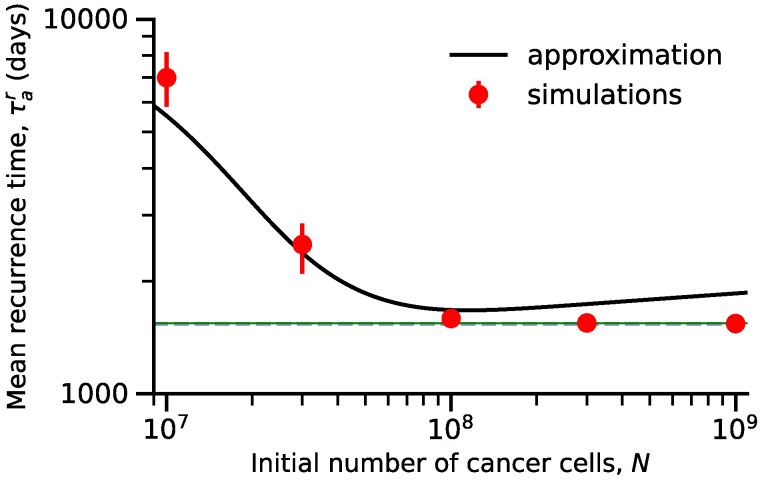
Tumor size decreases the mean recurrence time. The mean time for the mutant cell population to reach size *N*, the initial number of cancer cells. Our inhomogeneous Poisson-process approximation (solid black line, equation (D1)) is in agreement with simulation results (red markers with error bars for 95% CI) for intermediate size *N*. Simulation results converge to equation (D4) (blue dashed line) for large values of *N*. The green line represents the numerical solution of equation (D3). Parameters: $\lambda_{s}=0.1,\lambda_{a}=0.0899,$  $\lambda_{m}=0.1,$  $\mu_{s}=0.14,\mu_{a}=0.09,\mu_{m}=0.09,$  $u=10^{-2},v=10^{-7}$.

Figure 7 and Supplementary Fig. 7 show the agreement between our approximations and simulation results. For small tumors ($N\ll N_{a}^{*}$), the mean recurrence time can be approximated as the sum of the mean time for the first rescue mutation to appear ($\tau_{a}$) and the additional mean time for its lineage to reach size *N*. This additional time is the equivalent of Orr and Unckless (2014)’s “$t_{\mathrm{return}}$” (their equation 23). It grows logarithmically with tumor size *N* and may be the same order of magnitude as the mean evolutionary rescue time. Increasing the mutant growth rate, $r_{m}$, decreases the recurrence time, while increasing the sensitive and aneuploid growth rates, $r_{s}$ and $r_{a}$, respectively, increases the recurrence time.

For large tumors ($N\gg N_{m}^{*}$), the dynamics of the number of mutant cells is deterministic, and the mean recurrence time becomes independent of the initial tumor size *N*. Increasing either the mutant growth rate, $r_{m}$, or the mutation rate, *v*, decreases the time for the tumor to rebound to its initial size. Drugs that significantly increase the death rate of sensitive cells, $\mu_{s}$, but do not affect their division rate, $\lambda_{s}$, delay cancer recurrence. Drugs that decrease the sensitive division rate delay cancer recurrence time even more, because they effectively decrease the mutation rate (assuming mutations occur during division). Patients treated with such drugs may require a longer period of monitoring to guarantee the effectiveness of the treatment.

We note that, for small and large tumors, when $N\ll N_{a}^{*}$ or $N\gg N_{m}^{*}$, the asymptotic expressions for the mean recurrence time are independent of the chromosome missegregation rate *u*, and therefore, the rate at which the drug induces aneuploidy has no effect on the time for the tumor to rebound to its initial size *N*.

Appendix F gives us the probability that a mutant cancer cell population has not reached size *N* by time *t*. Figure 6b shows agreement between our approximations and simulation results for various values of *N*. Additionally, we derive the distribution of the recurrence time for a small tumor with $N=10^{6}$ cells, noting that the distribution is wide and right-skewed (Supplementary Fig. 4). It is highly unlikely to observe the recurrence of cancer at times smaller than $\frac{1}{r_{m}}\log\;\frac{r_{m}-r_{s}}{v\lambda_{s}}\approx 1500$ days for the parameter values in Table 1 with $\lambda_{a}=0.0899$, $\mu_{s}=0.14$, and $v=10^{-7}$ and independent of initial tumor size *N* (Fig. 6b).

The detection time $\tau_{a}^{M}$ is defined as the time for the tumor size to reach detection threshold *M*. We derive the mean detection time for $M=10^{7}$ in Appendix D. We find that for small- and intermediate-sized tumors the mean detection time is approximately equal to the mean recurrence time (i.e. $\tau_{a}^{r}\approx \tau_{a}^{M}$ for $N<N_{m}^{*}$). For large tumors, the mean detection time $\tau_{a}^{M}$ decreases logarithmically with tumor size *N*, while the recurrence time $\tau_{a}^{r}$ is constant (Supplementary Fig. 8). For large tumors, we also have $M<N_{m}^{*}<N$, so the mean detection time is shorter compared with the mean recurrence time, that is, the resistant tumor may be detected before recovering back to its initial size.

## Discussion

We have modeled a tumor—a population of cancer cells—exposed to drug treatment that causes it to decline in size toward potential extinction. In this scenario, the tumor can be “evolutionarily rescued” or escape extinction via two paths. In the direct path, a drug-sensitive cell acquires a mutation or aneuploidy that confers resistance and allows it to grow rapidly. In the indirect path, a sensitive cell first becomes aneuploid, which diminishes the drug’s effect, and then an aneuploid cell acquires a mutation that confers resistance (Fig. 1).

Using multitype branching processes, we derived the probability of evolutionary rescue of the tumor under the effects of aneuploidy, ranging from tolerance to partial resistance. We obtained exact and approximate expressions for the probability of evolutionary rescue (equation (1)). Our results show that the probability of evolutionary rescue increases with the initial tumor size *N*, the drug-sensitive growth rate $r_{s}$, the mutation rate *v*, and the aneuploidy rate *u*. Notably, the latter indicates that aneuploidy, even when it only provides tolerance, increases the probability that the tumor will be rescued, as long as it occurs frequently enough (Fig. 3a, equation (4)).

When aneuploid cells are partially resistant to the drug ($r_{s}\ll 0\ll r_{a}\ll r_{m}$), aneuploidy itself rescues the population (Fig. 4a). When aneuploidy only provides tolerance to the drug ($r_{s}\ll r_{a}\ll 0\ll r_{m}$), it cannot rescue the population. Instead, if the rate of occurrence of aneuploidy is high enough (equation (4)) then aneuploidy may act as a “stepping stone” through which the resistant mutant can appear more easily, given that the number of aneuploid cells declines slower than the number of drug-sensitive cells (Fig. 2). In this scenario, aneuploidy provides two advantages. First, it delays the extinction of the population, providing more time for the appearance of a resistance mutation. Second, it increases the population size relative to a drug-sensitive population, providing more cells in which mutations can occur. Together, this increases the cumulative number of mutants that arise (i.e. $Nuv\lambda_{s}\lambda_{a}/|r_{s}r_{a}|$).

We find that aneuploidy can significantly affect evolutionary rescue by reducing the threshold tumor size by several orders of magnitude, even when aneuploidy only provides tolerance, provided that the aneuploidy rate is high enough, $\lambda_{a}u>|r_{a}|$ (Fig. 3a, equation (4)). When the number of cells in the tumor is large enough (i.e. $N\gg N_{m}^{*}\approx 4\times 10^{7}$), a resistance mutation will occur in drug-sensitive cells before these cells become extinct. Therefore, large tumors are likely to be rescued with or without aneuploidy. Anti-cancer drugs are often used as adjuvant therapy after resection, in which case the number of cells in the tumor may be below the detection threshold of $∼10^{7}$ (Bozic *et al.* 2013). In these cases, aneuploidy can have a crucial role in the evolutionary rescue of the tumor and, therefore, in cancer recurrence. Indeed, secondary tumors are estimated to cause the majority of cancer-related deaths (Chaffer and Weinberg 2011). The importance of aneuploidy in the evolutionary rescue of secondary tumors is reinforced by the fact that metastases have been shown to have a chromosome missegregation rate two to three orders of magnitude higher compared with primary tumors (Kimmel *et al.* 2023).

As an example, we have parameterized our model using estimates from three triple-negative breast cancer (TNBC) clones under drug therapy. We find that TNBC clones are either in the first scenario, in which aneuploidy provides a partial or full resistance to the drug, or the third scenario, in which aneuploidy provides tolerance to the drug. It remains to be seen which tumor type and drug combinations produce stationary aneuploidies. Comparing the probability of evolutionary rescue between different tumors (Fig. 3b) suggests that some TNBC clones have a much higher probability of relapsing compared with melanoma and other TNBC clones. Notably, the probability of relapse in TNBC patients is >50% in the first 3–5 years after diagnosis (Taushanova *et al.* 2023), whereas in melanoma patients it is approximately in the first 5 years (Von Schuckmann *et al.* 2019; Wan *et al.* 2022).

Given that the mean time for secondary tumors to adapt to anticancer drugs can be of the order of 1,000 days (Supplementary Fig. 2a), aneuploidy can explain the reappearance of cancer even after initial remission. The theoretical prediction for the mean rescue time of tumors $<10^{8}$ cells is >4 years, consistent with previous estimates of the recurrence time of tumors after resection (Avanzini and Antal 2019). We found that aneuploidy complements evolutionary rescue through direct mutation because it produces rescue mutations mostly after the number of sensitive cells has decreased to a point where a direct mutation is no longer a feasible option for evolutionary rescue (Fig. 6a).

We hypothesized that standing genetic variation (the existence of aneuploid cancer cells in the tumor before the onset of therapy) could facilitate evolutionary rescue by reducing the waiting time for the appearance of aneuploid cells. We found that a drug that reduces the sensitive growth rate and does not significantly increase the chromosome missegregation rate will likely lead to evolutionary rescue through standing genetic variation (Fig. 5 and equation (5)). If the fraction of tumor cells that have the beneficial aneuploidy is $f\gg u\lambda_{s}/|r_{s}|\approx 2.5\%$, then evolutionary rescue is more likely to occur via standing variation rather than through de novo aneuploidy (Supplementary Fig. 5). For the parameter values, we focus on in our examples (Table 1), this fraction is an order of magnitude lower, and therefore, we expect evolutionary rescue to occur primarily by de novo aneuploidy.

Similar to Osmond *et al.* (2020), we find that two-step rescues can be more likely than simple one-step rescues, because the mutation rate to stepping-stone (aneuploid) genotypes can be much higher than that to fully resistant genotypes. The “stationary” scenario, in which the aneuploid growth rate is close to zero in the presence of the drug, is particularly interesting. Although this may seem like a small region of parameter space in a general model of evolutionary rescue (Fig. 4a, inset), it could be biologically significant for some tumors under specific drug therapies. Our results suggest that identifying aneuploidies that are tolerant or stationary may be worthwhile, as they may enhance the probability of rescue (Fig. 3a) and extend the window of opportunity for rescue (Fig. 6).

Experiments could test our model predictions. For example, to assess the effect of initial tumor size on the probability of evolutionary rescue, a large culture mass can be propagated from a single cancer cell in permissive conditions and then diluted to a range of starting tumor sizes. Then, the extinction or survival of these tumors can be monitored during exposure to anticancer drugs that induce aneuploidy or to saline solution for control (Ippolito *et al.* 2021). We can then compare the results of these experiments to predictions of our model to see if tumors with initial size below the threshold (equation (3)) are more likely to become extinct due to drug exposure. It may also be interesting to look for the involvement of aneuploidy in evolutionary rescue in other biological systems, such as evolution of yeast populations under different stress conditions (Pompei and Cosentino Lagomarsino 2023; Kohanovski *et al.* 2024).

Our model neglects the possibility of back-mutations from aneuploidy to euploidy, and also the possibility that the fitness of a euploid cell with a resistance mutation may be higher than that of an aneuploid cell with the same mutation. Both of these would be expected to reduce the importance of two-step rescues via aneuploidy. But for the former, unless the rate of loss of extra chromosomes is extremely high (for estimates in yeast, see Hose *et al.* 2024), comparable in magnitude to the growth rate $r_{a}$, we expect that its effect on the dynamics will be negligible. The latter possibility, in which aneuploidy substantially reduces the mutant growth rate, seems more likely to have an effect. Including it would be a straightforward extension to our model. In this case, it could actually be more important to consider the possibility of the loss of aneuploidy, as one would need to check the relative rates of the simple *sensitive*  →  *euploid mutant* path and the three-step *sensitive*  →  *aneuploid*  →  *aneuploid mutant*  →  *euploid mutant* path (Kohanovski *et al.* 2024).

We have assumed that cancer cell lineages are independent and have verified that this is accurate under simple logistic growth. This assumption neglects the potential effects of spatial structure and local interactions, which may be important in solid tumors. Such tumors can be spatially heterogeneous, with different genotypes inhabiting cellular niches and immune infiltration impacting growth in affected regions (Galon *et al.* 2010; Varrone *et al.* 2024). This can potentially impact the probability of evolutionary rescue (Martens *et al.* 2011).

### Conclusions

Our results quantitatively suggest that aneuploidy can play an important role in tumor adaptation to anticancer drugs when the tumor size is small or intermediate. Large tumors are predicted to adapt to anticancer drugs through direct mutation. In contrast, smaller tumors are predicted to become resistant directly by aneuploidy or by a resistance mutation that occurs in aneuploid cells that serve as evolutionary “stepping stones.” Thus, therapies that increase the rate of aneuploidy in tumors to combat cancer may also promote drug resistance.

## Supplementary Material

iyaf098_Supplementary_Data

## Data Availability

The code necessary to reproduce the results and the plots presented in this paper is available at https://github.com/yoavram-lab/EvolutionaryRescue. Supplemental material available at GENETICS online.

## Appendix A: Survival probability of a single lineage

To analyze evolutionary rescue in our model, we use the framework of *multi-type branching processes* (Harris 1963; Weissman *et al.* 2009). In the case of cancer, where all reproduction is binary fission, these branching processes are also *birth–death processes* (Kendall 1948). This allows us to find explicit expressions for the *survival probability*: the probability that a lineage descended from a single cell does not become extinct.

Let $p_{s}$, $p_{a}$, and $p_{m}$ be the survival probabilities of a population consisting initially of single sensitive cell, aneuploid cell, or mutant cell, respectively. The complements $1-p_{s}$, $1-p_{a}$, and $1-p_{m}$ are the extinction probabilities, which satisfy each its respective equation (Harris 1963),

(A1 )
$$\begin{array}{rl} 1-p_{s} & =\frac{\mu_{s}}{\lambda_{s}+\mu_{s}+u\lambda_{s}+v\lambda_{s}}\\ & \;+\frac{u\lambda_{s}}{\lambda_{s}+\mu_{s}+u\lambda_{s}+v\lambda_{s}}\left(1-p_{a}\right)\left(1-p_{s}\right)\\ & \;+\frac{\lambda_{s}}{\lambda_{s}+\mu_{s}+u\lambda_{s}+v\lambda_{s}}{\left(1-p_{s}\right)}^{2}\\ & \;+\frac{v\lambda_{s}}{\lambda_{s}+\mu_{s}+u\lambda_{s}+v\lambda_{s}}\left(1-p_{m}\right)\left(1-p_{s}\right),\\ 1-p_{a} & =\frac{\mu_{a}}{\lambda_{a}+\mu_{a}+v\lambda_{a}}+\frac{v\lambda_{a}}{\lambda_{a}+\mu_{a}+v\lambda_{a}}\left(1-p_{m}\right)\left(1-p_{a}\right)\\ & \;+\frac{\lambda_{a}}{\lambda_{a}+\mu_{a}+v\lambda_{a}}{\left(1-p_{a}\right)}^{2},\\ 1-p_{m} & =\frac{\mu_{m}}{\lambda_{m}+\mu_{m}}+\frac{\lambda_{m}}{\lambda_{m}+\mu_{m}}{\left(1-p_{m}\right)}^{2}. \end{array}$$

The survival probabilities are given by the smallest solution for each quadratic equation (Uecker *et al.* 2015). Therefore, we have

(A2 )
$$\begin{array}{rl} \;p_{s} & =\frac{\lambda_{s}-\mu_{s}-u\lambda_{s}p_{a}-v\lambda_{s}p_{m}}{2\lambda_{s}}\\ & \;+\frac{\sqrt{{\left(\lambda_{s}-\mu_{s}-u\lambda_{s}p_{a}-v\lambda_{s}p_{m}\right)}^{2}\;+\;4\lambda_{s}^{2}\left(up_{a}+vp_{m}\right)}}{2\lambda_{s}},\\ p_{a} & =\frac{\lambda_{a}-\mu_{a}-v\lambda_{a}p_{m}+\sqrt{{\left(\lambda_{a}-\mu_{a}-v\lambda_{a}p_{m}\right)}^{2}+4\lambda_{a}^{2}vp_{m}}}{2\lambda_{a}},\\ p_{m} & =\frac{\lambda_{m}-\mu_{m}}{\lambda_{m}}. \end{array}$$

Note that the equation for $p_{s}$ depends on both $p_{a}$ and $p_{m}$, and the equation for $p_{a}$ depends on $p_{m}$. To proceed, we can plug the solution for $p_{m}$ and $p_{a}$ into the solution for $p_{s}$. We perform this for three different scenarios.

### Scenario 1: Aneuploid cells are partially resistant

We first assume that aneuploidy provides partial resistance to drug therapy, $\lambda_{a}>\mu_{a}$, and that this resistance is significant, $(\lambda_{a}-\mu_{a}-v\lambda_{a}p_{m}{)}^{2}>4\lambda_{a}^{2}vp_{m}$. We thus rewrite equation (A2) as

$$\begin{array}{rl} \;p_{s} & =\frac{\lambda_{s}-\mu_{s}-u\lambda_{s}p_{a}-v\lambda_{s}p_{m}}{2\lambda_{s}}\\ & \;\times \left(1-\sqrt{1+\frac{4\lambda_{s}^{2}\left(vp_{m}+up_{a}\right)}{{\left(\lambda_{s}-\mu_{s}-u\lambda_{s}p_{a}-v\lambda_{s}p_{m}\right)}^{2}}}\right),\;\text{and}\\ p_{a} & =\frac{\lambda_{a}-\mu_{a}-v\lambda_{a}p_{m}}{2\lambda_{a}}\left(1+\sqrt{1+\frac{4\lambda_{a}^{2}vp_{m}}{{\left(\lambda_{a}-\mu_{a}-v\lambda_{a}p_{m}\right)}^{2}}}\right). \end{array}$$

Using the Taylor expansion $\sqrt{1+x}=1+x/2+O(x^{2})$ and assuming u,v≪1, we obtain the following approximation for the survival probability of a population initially consisting of a single sensitive cell,

(A3 )
$$p_{s}\approx -\frac{v\lambda_{s}p_{m}+u\lambda_{s}p_{a}}{\lambda_{s}-\mu_{s}-u\lambda_{s}p_{a}-v\lambda_{s}p_{m}}$$

(A4 )
$$\begin{array}{rl} & \approx -\frac{1}{\lambda_{s}-\mu_{s}}[\frac{u\lambda_{s}\left(\lambda_{a}-\mu_{a}\right)}{\lambda_{a}}+\frac{uv\lambda_{s}\lambda_{a}\left(\lambda_{m}-\mu_{m}\right)}{\lambda_{m}\left(\lambda_{a}-\mu_{a}\right)}\\ & \;+\frac{v\lambda_{s}\left(\lambda_{m}-\mu_{m}\right)}{\lambda_{m}}]. \end{array}$$

Now uv is small, and if we use the fact that v≪u, we have:

(A5 )
$$p_{s}\approx \frac{u\lambda_{s}}{\left|r_{s}\right|}\frac{r_{a}}{\lambda_{a}}.$$

However, if aneuploidy is sufficiently rare such that

$$\begin{array}{rl} \frac{u\lambda_{s}r_{a}}{\lambda_{a}}<\frac{v\lambda_{s}r_{m}}{\lambda_{m}} & \Rightarrow u\lambda_{a}<\frac{v\lambda_{a}^{2}r_{m}}{\lambda_{m}}\frac{1}{r_{a}}<\frac{v\lambda_{a}^{2}r_{m}}{\lambda_{m}}\frac{1}{\sqrt{4\lambda_{a}^{2}vp_{m}}}\\ & \Rightarrow u\lambda_{a}<T^{*}, \end{array}$$

where $T^{*}=(4v\lambda_{a}^{2}r_{m}/\lambda_{m}{)}^{-1/2}$ and in the second inequality we used the fact that $r_{a}^{2}>4\lambda_{a}^{2}vp_{m}$. In this scenario, adaptation is through direct mutation and:

$$p_{s}\approx \frac{v\lambda_{s}}{\left|r_{s}\right|}\frac{r_{m}}{\lambda_{m}}.$$

### Scenario 2: Aneuploid cells are tolerant

We now assume that aneuploidy provides tolerance to drug therapy, that is, the number of aneuploid cells significantly declines over time, but at a lower rate than the number of sensitive cells, $\lambda_{s}-\mu_{s}<\lambda_{a}-\mu_{a}<0$. We also assume that the decline is significant, $(\lambda_{a}-\mu_{a}-v\lambda_{a}p_{m}{)}^{2}>4\lambda_{a}^{2}vp_{m}$. We rewrite equation (A2) as

(A6 )
$$\begin{array}{rl} \;p_{s} & =\frac{\lambda_{s}-\mu_{s}-u\lambda_{s}p_{a}-v\lambda_{s}p_{m}}{2\lambda_{s}}\\ & \;\times \left(1-\sqrt{1+\frac{4\lambda_{s}^{2}\left(vp_{m}+up_{a}\right)}{{\left(\lambda_{s}-\mu_{s}-u\lambda_{s}p_{a}-v\lambda_{s}p_{m}\right)}^{2}}}\right),\\ p_{a} & =\frac{\lambda_{a}-\mu_{a}-v\lambda_{a}p_{m}}{2\lambda_{a}}\left(1-\sqrt{1+\frac{4\lambda_{a}^{2}vp_{m}}{{\left(\lambda_{a}-\mu_{a}-v\lambda_{a}p_{m}\right)}^{2}}}\right). \end{array}$$

Since u,v≪1, the term in the root can be approximated using a Taylor expansion. So, substituting the expressions for $p_{a}$ and $p_{m}$, we have

(A7 )
$$\begin{array}{rl} \;p_{s} & \approx -\frac{v\lambda_{s}p_{m}+u\lambda_{s}p_{a}}{\lambda_{s}-\mu_{s}-u\lambda_{s}p_{a}-v\lambda_{s}p_{m}}\\ & \approx \frac{1}{\lambda_{s}-\mu_{s}-u\lambda_{s}p_{a}-v\lambda_{s}p_{m}}\left[\frac{uv\lambda_{s}\lambda_{a}\left(\lambda_{m}-\mu_{m}\right)}{\lambda_{m}\left(\lambda_{a}-\mu_{a}-v\lambda_{a}\right)}-\frac{v\lambda_{s}\left(\lambda_{m}-\mu_{m}\right)}{\lambda_{m}}\right]\\ & \approx \frac{v\lambda_{s}\left(\lambda_{m}-\mu_{m}\right)}{\lambda_{m}\left(\lambda_{s}-\mu_{s}\right)}\left[\frac{u\lambda_{a}}{\left(\lambda_{a}-\mu_{a}\right)}-1\right]\\ & =\frac{v\lambda_{s}r_{m}}{\lambda_{m}|r_{s}|}\left(\frac{u\lambda_{a}}{\left|r_{a}\right|}+1\right). \end{array}$$

If we assume that aneuploidy is not rare ($u\lambda_{a}>|r_{a}|$) then we have:

(A8 )
$$p_{s}\approx \frac{u\lambda_{s}}{\left|r_{s}\right|}\frac{v\lambda_{a}}{\left|r_{a}\right|}\frac{r_{m}}{\lambda_{m}}.$$

### Scenario 3: Aneuploid cells are stationary

We now assume that the growth rate of aneuploid cells is close to zero (either positive or negative), such that $(r_{a}-v\lambda_{a}p_{m}{)}^{2}\ll 4\lambda_{a}^{2}vp_{m}$. We rewrite equation (A2) as

(A9 )
$$p_{a}=\frac{\lambda_{a}-\mu_{a}-v\lambda_{a}p_{m}+2\sqrt{\lambda_{a}^{2}vp_{m}}{\left(1+\frac{{\left(\lambda_{a}-\mu_{a}-v\lambda_{a}p_{m}\right)}^{2}}{4\lambda_{a}^{2}vp_{m}}\right)}^{\frac{1}{2}}}{2\lambda_{a}}.$$

Using a following Taylor series expansion for small $(\lambda_{a}-\mu_{a}-v\lambda_{a}p_{m}{)}^{2}/4\lambda_{a}^{2}vp_{m}$,

$${\left(1+\frac{{\left(\lambda_{a}-\mu_{a}-v\lambda_{a}p_{m}\right)}^{2}}{4\lambda_{a}^{2}vp_{m}}\right)}^{\frac{1}{2}}=1+\frac{{\left(\lambda_{a}-\mu_{a}-v\lambda_{a}p_{m}\right)}^{2}}{8\lambda_{a}^{2}vp_{m}}+\cdots ,$$

we obtain the approximation

(A10 )
$$\begin{array}{rl} \;p_{a} & \approx \frac{\lambda_{a}-\mu_{a}-v\lambda_{a}p_{m}+2\sqrt{\lambda_{a}^{2}vp_{m}}\left[1+\frac{{\left(\lambda_{a}-\mu_{a}-v\lambda_{a}p_{m}\right)}^{2}}{8\lambda_{a}^{2}vp_{m}}\right]}{2\lambda_{a}}\\ & =\frac{\lambda_{a}-\mu_{a}-v\lambda_{a}p_{m}+2\sqrt{\lambda_{a}^{2}vp_{m}}+\frac{{\left(\lambda_{a}-\mu_{a}-v\lambda_{a}p_{m}\right)}^{2}}{4\sqrt{\lambda_{a}^{2}vp_{m}}}}{2\lambda_{a}}\\ & =\frac{{\left(\lambda_{a}-\mu_{a}-v\lambda_{a}p_{m}+2\sqrt{\lambda_{a}^{2}vp_{m}}\right)}^{2}+4\lambda_{a}^{2}vp_{m}}{8\lambda_{a}\sqrt{\lambda_{a}^{2}vp_{m}}}\\ & =\frac{4\lambda_{a}^{2}vp_{m}+4\lambda_{a}^{2}vp_{m}{\left(1+\frac{\lambda_{a}-\mu_{a}-v\lambda_{a}p_{m}}{2\sqrt{\lambda_{a}^{2}vp_{m}}}\right)}^{2}}{8\lambda_{a}\sqrt{\lambda_{a}^{2}vp_{m}}}\\ & =\frac{1}{2\lambda_{a}}\left(\lambda_{a}-\mu_{a}-v\lambda_{a}p_{m}+2\sqrt{\lambda_{a}^{2}vp_{m}}\right). \end{array}$$

Plugging this in equation (A3), the survival probability of a population starting from one sensitive cell is

(A11 )
$$\begin{array}{rl} \;p_{s} & \approx -\frac{1}{\lambda_{s}-\mu_{s}-u\lambda_{s}p_{a}-v\lambda_{s}p_{m}}[v\lambda_{s}\frac{\lambda_{m}-\mu_{m}}{\lambda_{m}}\\ & \;+\frac{u\lambda_{s}}{2\lambda_{a}}\left(\lambda_{a}-\mu_{a}-v\lambda_{a}p_{m}+2\sqrt{\lambda_{a}^{2}vp_{m}}\right)]\\ & =-\frac{1}{\lambda_{s}-\mu_{s}-u\lambda_{s}p_{a}-v\lambda_{s}p_{m}}[v\lambda_{s}\frac{\lambda_{m}-\mu_{m}}{\lambda_{m}}\\ & \;+\frac{u\lambda_{s}}{2\lambda_{a}}\left(\lambda_{a}-\mu_{a}-v\lambda_{a}p_{m}\right)+u\lambda_{s}\sqrt{\frac{v\left(\lambda_{m}-\mu_{m}\right)}{\lambda_{m}}}]\\ & \approx -\frac{1}{r_{s}}\left[v\lambda_{s}\frac{r_{m}}{\lambda_{m}}+\frac{u\lambda_{s}\left(r_{a}-v\lambda_{a}p_{m}\right)}{2\lambda_{a}}+u\lambda_{s}\sqrt{\frac{vr_{m}}{\lambda_{m}}}\right]. \end{array}$$

Using the fact that

$${\left(r_{a}-v\lambda_{a}p_{m}\right)}^{2}\ll 4\lambda_{a}^{2}vp_{m}\Rightarrow \frac{r_{a}-v\lambda_{a}p_{m}}{2\lambda_{a}}\ll \sqrt{\frac{v\lambda_{a}r_{m}}{\lambda_{m}}},$$

and v≪u we obtain:

(A12 )
$$p_{s}\approx \frac{u\lambda_{s}}{\left|r_{s}\right|}\sqrt{\frac{v\lambda_{a}r_{m}}{\lambda_{m}}}.$$

## Appendix B: Evolutionary rescue probability

Using the fact that $r_{a}-v\lambda_{a}p_{m}\approx r_{a}$ we write the condition $(r_{a}-v\lambda_{a}p_{m}{)}^{2}\ll 4\lambda_{a}^{2}vp_{m}$ as:

$$r_{a}^{2}\ll 4\lambda_{a}^{2}vp_{m}\Rightarrow -1\ll r_{a}T^{*}\ll 1,$$

where $T^{*}=(4v\lambda_{a}^{2}r_{m}/\lambda_{m}{)}^{-1/2}$. Substituting equations (A5), (A8), and (A12) into equation (1), the evolutionary rescue probability can be approximated by

(B1 )
$$\begin{array}{rl} & p_{\mathrm{rescue}}\\ & \;\approx \left\{ \begin{array}{cc} 1-\exp\;\left[-\frac{u\lambda_{a}}{\left|r_{s}\right|}\frac{v\lambda_{s}}{\left|r_{a}\right|}\frac{r_{m}}{\lambda_{m}}N\right],\; & r_{a}T^{*}\ll -1,\\ 1-\exp\;\left[-\frac{u\lambda_{s}}{\left|r_{s}\right|}\sqrt{\frac{v\lambda_{a}r_{m}}{\lambda_{m}}}N\right],\; & -1\ll r_{a}T^{*}\ll 1,\\ 1-\exp\;\left[-\frac{u\lambda_{s}}{\left|r_{s}\right|}\frac{r_{a}}{\lambda_{a}}N\right],\; & 1\ll r_{a}T^{*}. \end{array}\right. \end{array}$$

## Appendix C: Evolutionary rescue time

We first calculate the expected time for the appearance of the first mutant that rescues the cell population. This can occur either through the evolutionary trajectory *sensitive*→*mutant* or through the trajectory *sensitive*→*aneuploid*→*mutant*. We start with the former.

Assuming no aneuploidy (u=0), we define $T_{m}$ to be the time at which the first mutant cell appears that will avoid extinction and will therefore rescue the population. Note that if extinction occurs, that is the frequency of mutants after a long time is zero, $m_{\infty }=0$, then it is implied that $T_{m}=\infty$, and vice versa if $T_{m}<\infty$ then $m_{\infty }>0$.

The number of successful mutants generated until time *t* can be approximated by an inhomogeneous Poisson process with rate $R_{m}(t)=v\lambda_{s}p_{m}s_{t}$, where $s_{t}=Ne^{r_{s}t}$ is the number of sensitive cells at time *t*. Note that

(C1 )
$$\int_{0}^{t}R_{m}(z)\;dz=v\lambda_{s}p_{m}N\frac{\exp\;[r_{s}t]-1}{r_{s}}\approx v\lambda_{s}p_{m}Nt,$$

by integrating the exponential and because $\frac{1}{r_{s}}(\exp\;[r_{s}t]-1)=t+O\left(r_{s}t^{2}\right)$. The probability density function of $T_{m}$ is thus $R_{m}(t)\exp\;(-\int_{0}^{t}R_{m}(z)\;dz)$ (Allen 2010). Therefore, the probability density function of the conditional random variable $\left(T_{m}|T_{m}<\infty \right)$ is $f_{m}(t)=R_{m}(t)\exp\;(-\int_{0}^{t}R_{m}(z)\;dz)/p_{\mathrm{rescue}}$.

We are interested in the mean conditional time, $\tau_{m}=E[T_{m}|T_{m}<\infty ]$, which is given by

(C2 )
$$\tau_{m}=\int_{0}^{\infty }tf_{m}(t)\;dt=\frac{\int_{0}^{\infty }tR_{m}(t)\exp\;\left(-\int_{0}^{t}R_{m}(z)\;dz\right)\;dt}{p_{\mathrm{rescue}}},$$

Therefore, plugging equations (1) and (C1) in equation (C2),

(C3 )
$$\tau_{m}=\int_{0}^{\infty }tv\lambda_{s}Ne^{r_{s}t}\frac{e^{-v\lambda_{s}Np_{m}\frac{e^{r_{s}t}-1}{r_{s}}}}{1-{\left(1-p_{s}\right)}^{N}}\;dt\approx \int_{0}^{\infty }tv\lambda_{s}Ne^{r_{s}t}\frac{e^{-v\lambda_{s}Np_{m}t}}{1-e^{-Np_{s}}}\;dt.$$

Supplementary Fig. 2b shows the agreement between this approximation and simulation results.

Assuming aneuploidy is possible (u>0), we define $T_{a}$ to be the time at which the first mutant cell appears that will rescue the population. We are interested in the mean conditional time, $\tau_{a}=E[T_{a}|T_{a}<\infty ]$.

When $Nu\lambda_{s}/|r_{s}|\gg 1$ the aneuploid frequency dynamics is roughly deterministic and therefore can be approximated by

(C4 )
$$a_{t}\approx \frac{Nu\lambda_{s}}{r_{s}-r_{a}}\left(e^{r_{s}t}-e^{r_{a}t}\right).$$

As a result, the number of successful mutants created by direct mutation and via aneuploidy can be approximated by inhomogeneous Poisson processes with the rates

$$\begin{array}{rl} n_{1}\left(t\right) & =v\lambda_{a}p_{m}a_{t},\\ r_{2}\left(t\right) & =v\lambda_{s}p_{m}s_{t}, \end{array}$$

and the expected number of successful mutants created by direct mutation and via aneuploidy until time *t* is given by:

(C5 )
$$M_{a}\left(t\right)=v\lambda_{a}p_{m}\int_{0}^{t}a_{z}\;dz=\frac{uv\lambda_{s}\lambda_{a}Np_{m}}{r_{s}-r_{a}}\left(\frac{e^{r_{s}t}-1}{r_{s}}-\frac{e^{r_{a}t}-1}{r_{a}}\right),$$

(C6 )
$$M_{m}\left(t\right)=v\lambda_{s}p_{m}\int_{0}^{t}s_{z}\;dz=v\lambda_{s}Np_{m}\frac{e^{r_{s}t}-1}{r_{s}}.$$

For large initial population sizes, we assume that the two processes are independent and as a result, they can be merged into a single Poisson process with rate $R_{a}(t)=(n_{1}+r_{2})(t)$. The mean time to the appearance of the first rescue mutant is

(C7 )
$$\begin{array}{rl} \tau_{a} & =\frac{\int_{0}^{\infty }tR_{a}(t)\exp\;\left(-\int_{0}^{t}R_{a}(z)\;dz\right)\;dt}{p_{\mathrm{rescue}}}=\int_{0}^{\infty }t\left(v\lambda_{a}p_{m}a_{t}+v\lambda_{s}p_{m}s_{t}\right)\\ & \times \frac{\exp\;\left[-\frac{uv\lambda_{s}\lambda_{a}Np_{m}}{r_{s}-r_{a}}\left(\frac{e^{r_{s}t}-1}{r_{s}}-\frac{e^{r_{a}t}-1}{r_{a}}\right)-v\lambda_{s}Np_{m}\frac{e^{r_{s}t}-1}{r_{s}}\right]}{\left(1-e^{-Np_{s}}\right)}\;dt, \end{array}$$

which we plot in Supplementary Fig. 2a as a function of the initial population size, *N*.

Paradoxically, we observe from Supplementary Fig. 2 that the mean time of a rescue mutation to appear is significantly shorter for the scenario when u=0 when compared with the scenario u>0; however, this can be explained by the fact this mean time is conditioned on evolutionary rescue and, as a result, aneuploidy increase the *window of opportunity* in which a rescue mutation could appear thus increasing the mean time as well (Fig. 2).

Let $N_{a}^{*}$ and $N_{m}^{*}$ be the threshold population size above which evolutionary rescue through aneuploidy or direct mutation, respectively, is likely. The mean time $\tau_{a}$ can be written as:

$$\begin{array}{rl} \tau_{a} & =\int_{0}^{\infty }tf(t)\;dt=\int_{0}^{\infty }t\frac{d}{dt}F(t)\;dt=-\int_{0}^{\infty }t\frac{d}{dt}S(t)\;dt\\ & =-{\left[tS(t)\right]}_{0}^{\infty }+\int_{0}^{\infty }S(t)\;dt\\ & =\int_{0}^{\infty }S(t)\;dt, \end{array}$$

where f(t), F(t), and S(t) are the probability density function, cumulative distribution function, and survival function. In our case, $S(t)=\exp\;(-\int_{0}^{t}R_{a}(z)\;dz)$. Additionally, for $N\gg N_{m}^{*}$ we have $1-e^{-Np_{s}}\approx 1$ and, as a result, we have:

$$\begin{array}{rl} \tau_{a} & =\int_{0}^{\infty }e^{-\int_{0}^{t}R_{a}\left(z\right)\;dz}\;dt\\ & =\int_{0}^{\infty }\exp\;[-\frac{uv\lambda_{s}\lambda_{a}Np_{m}}{r_{s}-r_{a}}\left(\frac{e^{r_{s}t}-1}{r_{s}}-\frac{e^{r_{a}t}-1}{r_{a}}\right)\\ & \;-v\lambda_{s}Np_{m}\frac{e^{r_{s}t}-1}{r_{s}}]\;dt, \end{array}$$

and we use the following Taylor series expansions:

$$\begin{array}{rl} \frac{e^{r_{s}t}-1}{r_{s}} & =\frac{1+r_{s}t+O(t^{2})-1}{r_{s}}=t+O(t^{2}).\\ \frac{e^{r_{a}t}-1}{r_{a}} & =\frac{1+r_{a}t+O(t^{2})-1}{r_{a}}=t+O(t^{2}), \end{array}$$

to obtain a simpler approximation for $\tau_{a}$:

(C8 )
$$\tau_{a}\approx \int_{0}^{\infty }e^{-v\lambda_{s}Np_{m}t}\;dt=\frac{1}{v\lambda_{s}Np_{m}}.$$

If $N\ll N_{a}^{*}$ then the probability distribution of evolutionary rescue time is skewed toward small values (i.e. evolutionary rescue occurs conditioned on it happening). As a result, we have:

$$\begin{array}{rl} \frac{e^{r_{s}t}-1}{r_{s}} & =\frac{1+r_{s}t+O\left({\left(r_{s}t\right)}^{2}\right)-1}{r_{s}}=t+O\left(r_{s}t^{2}\right)\\ \frac{e^{r_{a}t}-1}{r_{a}} & =\frac{1+r_{a}t+O\left({\left(r_{a}t\right)}^{2}\right)-1}{r_{a}}=t+O\left(r_{a}t^{2}\right) \end{array}$$

We observe that the first term in the exponential in equation (C7) can be approximated to be zero and the second term as -$v\lambda_{s}Np_{m}t$ where t≪1. As a result, we can approximate:

$$\exp\;\left[-\frac{uv\lambda_{s}\lambda_{a}Np_{m}}{r_{s}-r_{a}}\left(\frac{e^{r_{s}t}-1}{r_{s}}-\frac{e^{r_{a}t}-1}{r_{a}}\right)-v\lambda_{s}Np_{m}\frac{e^{r_{s}t}-1}{r_{s}}\right]∼1$$

Additionally, since $N\ll N_{a}^{*}\ll N_{m}^{*}$, evolutionary rescue is more likely to occur through the trajectory sensitive→aneuploid→mutant. As a result, we can write Equation (C7) as:

(C9 )
$$\begin{array}{rl} \tau_{a} & \approx \frac{\int_{0}^{\infty }tv\lambda_{a}p_{m}a_{t}\;dt}{1-e^{-Np_{s}}}\approx \frac{uv\lambda_{a}\lambda_{s}p_{m}|r_{s}+r_{a}|}{p_{s}r_{a}^{2}r_{s}^{2}}=\frac{1}{\left|r_{s}\right|}+\frac{1}{\left|r_{a}\right|}, \end{array}$$

where in the last line we used the fact that $1/p_{s}=N_{a}^{*}$ and Equation (3).

If a fraction *f* of the cancer cells are aneuploid when the drug is administered then the expected number of successful mutants generated until time *t* is given by:

$$\begin{array}{rl} n_{1}^{f}\left(t\right) & =v\lambda_{a}p_{m}\int_{0}^{t}a_{z}\;dz\\ & =\left(1-f\right)\frac{uv\lambda_{s}\lambda_{a}Np_{m}}{r_{s}-r_{a}}\left(\frac{e^{r_{s}t}-1}{r_{s}}-\frac{e^{r_{a}t}-1}{r_{a}}\right)\\ & \;+fv\lambda_{a}Np_{m}\frac{e^{r_{a}t}-1}{r_{a}},\\ r_{2}^{f}\left(t\right) & =v\lambda_{s}p_{m}\int_{0}^{t}s_{z}\;dz\\ & =\left(1-f\right)v\lambda_{s}Np_{m}\frac{e^{r_{s}t}-1}{r_{s}}, \end{array}$$

and the mean evolutionary rescue time is given by:

(C10 )
$${\tilde{\tau }}_{a}=\frac{\int_{0}^{\infty }tR_{a}^{f}(t)\exp\;\left(-\int_{0}^{t}R_{a}^{f}(z)\;dz\right)\;dt}{p_{\mathrm{rescue}}},$$

where $R_{a}^{f}(t)=n_{1}^{f}(t)+r_{2}^{f}(t)$ and $p_{\mathrm{rescue}}=1-\exp\;[-(1-f)p_{s}N-fp_{a}N]$. We plot our approximation in Supplementary Fig. 9 together with simulated data.

## Appendix D: Recurrence time

In the following, we assume aneuploid cells are tolerant ($r_{a}T^{*}\ll -1$) and frequent enough to affect the evolution of drug resistance ($u\lambda_{a}\gg \max\left(-r_{a},1/T^{*}\right)$). We define the proliferation time $\tau_{a}^{p}$ to be the expected time for the number of resistant cells to grow to the initial tumor size *N*. The number of rescue lineages generated by the sensitive population is given by equation (C5) (see Supplementary Fig. 3),

$$n_{1}\left(\infty \right)=\frac{uv\lambda_{s}\lambda_{a}Np_{m}}{\left|r_{s}|\;|r_{a}\right|}+\frac{v\lambda_{s}Np_{m}}{\left|r_{s}\right|}=\frac{N}{N_{a}^{*}}+\frac{N}{N_{m}^{*}}.$$

We use equations (3) and (2) to write $N_{a}^{*}=\frac{\left|r_{s}|\;|r_{a}\right|}{uv\lambda_{s}\lambda_{a}p_{m}}$ and $N_{m}^{*}=\frac{\left|r_{s}\right|}{v\lambda_{s}p_{m}}$.

We distinguish between small, intermediate, or large tumors. In small ($N\ll N_{a}^{*}\ll N_{m}^{*}$) tumors, we have at most one lineage that rescues the cancer cell population. As a result, the recurrence time is given by (Avanzini and Antal 2019)

(D1 )
$$\tau_{a}^{r}\approx \tau_{a}+\frac{\log\;\left(\;p_{m}N\right)}{r_{m}}.$$

The factor $p_{m}$ in the second term of equation (D1) occurs because the lineage is conditioned to survive stochastic extinction and the time to reach *N* is shorter compared with the scenario where the lineage is not conditioned to survive stochastic extinction (Maynard Smith and Haigh 1974; Orr and Unckless 2014). Therefore, in small tumors, we can substitute equation (C9) for $\tau_{a}$ in equation (D1) to get (blue line in Supplementary Fig. 7)

$$\tau_{a}^{r}\approx 1/|r_{a}|+\log\;\left(\;p_{m}N\right)/r_{m}.$$

In intermediate tumors, $N_{a}^{*}\ll N\ll N_{m}^{*}$, the sensitive cell population generates a number of rescue lineages in a short period of time after $\tau_{a}$. The time it takes the rescue lineages to reach the initial population size *N* is small compared with the time it took the lineages to appear. Therefore, the mean recurrence time can be approximated by substituting equation (C7) for $\tau_{a}$ in equation (D1) (black line in Supplementary Fig. 7).

In large tumors, $N_{m}^{*}\ll N$, the sensitive population produces a large number of rescue lineages in a short period of time. As a result, the recurrence time is obtained by solving the following system of ODEs:

(D2 )
$$\begin{array}{rl} \frac{ds}{dt} & =r_{s}s,\\ \frac{da}{dt} & =r_{a}a+u\lambda_{s}s,\\ \frac{dm}{dt} & =r_{m}m+v\lambda_{a}a+v\lambda_{s}s. \end{array}$$

Solving the system of ODEs for initial condition (s(0),a(0),m(0))=(N,0,0) we obtain:

$$m(t)=\frac{Nuv\lambda_{a}\lambda_{s}}{r_{a}-r_{s}}\left[\frac{e^{r_{m}t}-e^{r_{a}t}}{r_{m}-r_{a}}-\frac{e^{r_{m}t}-e^{r_{s}t}}{r_{m}-r_{s}}\right]+Nv\lambda_{s}\frac{e^{r_{m}t}-e^{r_{s}t}}{r_{m}-r_{s}}.$$

We obtain $\tau_{a}^{r}$ by solving the above equation for time $t=\tau_{a}^{r}$ at which the number of mutant cells reaches *N*, that is, we solve $m(\tau_{a}^{r})=N$,

(D3 )
$$1=\frac{uv\lambda_{a}\lambda_{s}}{r_{a}-r_{s}}\left[\frac{e^{r_{m}\tau_{a}^{r}}-e^{r_{a}\tau_{a}^{r}}}{r_{m}-r_{a}}-\frac{e^{r_{m}\tau_{a}^{r}}-e^{r_{s}\tau_{a}^{r}}}{r_{m}-r_{s}}\right]+v\lambda_{s}\frac{e^{r_{m}\tau_{a}^{r}}-e^{r_{s}\tau_{a}^{r}}}{r_{m}-r_{s}}.$$

Equation (D3) is a transcendental equation that cannot be solved exactly but we can obtain an approximation by noting that the tumor size *N* is large, and because the sensitive and aneuploid cells have negative growth rates, almost all of the growth needed to rebound to size *N* will be by mutant cells. Mathematically, this corresponds to $e^{r_{m}\tau_{a}^{r}}$ being much larger than the other exponential terms, $e^{r_{s}\tau_{a}^{r}}$ and $e^{r_{a}\tau_{a}^{r}}$. Equation (D3) can then be rewritten as

$$1\approx \frac{v\lambda_{s}e^{r_{m}\tau_{a}^{r}}}{r_{m}-r_{s}}\left(\frac{u\lambda_{a}}{r_{m}-r_{a}}+1\right).$$

Assuming that rate of aneuploidy is not so high that it overwhelms the aneuploids’ growth disadvantage relative to the mutants ($u\lambda_{a}\ll r_{m}-r_{a}$), we can neglect the first term in parentheses above, yielding (green line in Supplementary Fig. 7)

(D4 )
$$\tau_{a}^{r}\approx \frac{1}{r_{m}}\log\;\left(\frac{r_{m}-r_{s}}{v\lambda_{s}}\right).$$

We emphasize that in this case the population is large enough to produce mutants without needing to pass through aneuploidy ($N\gg N_{m}^{*}$), and so the terms arising from the evolutionary trajectory *sensitive*  →  *aneuploid*  →  *mutant* do not contribute to the above approximation.

Additionally, we note that if we are interested in the time until the tumor reaches a detectable size *M* then our above analysis is valid but in equation (D1) we change

(D5 )
$$\tau_{a}^{r,M}\approx \tau_{a}+\frac{\log\;\left(\;p_{m}M\right)}{r_{m}},$$

and equation (D4) becomes

(D6 )
$$\tau_{a}^{r,M}\approx \frac{1}{r_{m}}\log\;\left(\frac{M\left(r_{m}-r_{s}\right)}{v\lambda_{s}N}\right),$$

which we plot in Supplementary Fig. 8 and observe that our approximations are in agreement with simulations.

## Appendix E: Distribution of evolutionary rescue time

The probability that a successful mutant has been generated by time *t* is given by:

$$\begin{array}{rl} P\left(\text{rescue},t\right) & =P\left(T_{a}<t\right)\\ & =1-\exp\;\left\{-\left[n_{1}\left(t\right)+r_{2}\left(t\right)\right]\right\}\\ & =1-\exp\;\{-[\frac{uv\lambda_{s}\lambda_{a}Np_{m}}{r_{s}-r_{a}}\left(\frac{e^{r_{s}t}-1}{r_{s}}-\frac{e^{r_{a}t}-1}{r_{a}}\right)\\ & \;+v\lambda_{s}Np_{m}\frac{e^{r_{s}t}-1}{r_{s}}]\}, \end{array}$$

where $T_{a}$ is the time at which the first mutant cell appears that will avoid extinction and which was defined in Appendix C.

As a result, the probability that a successful mutant has not been generated by time *t* is:

(E1 )
$$\begin{array}{rl} 1-P\left(\text{rescue},t\right) & =\exp\;\{-[\frac{uv\lambda_{s}\lambda_{a}Np_{m}}{r_{s}-r_{a}}\left(\frac{e^{r_{s}t}-1}{r_{s}}-\frac{e^{r_{a}t}-1}{r_{a}}\right)\\ & \;+v\lambda_{s}Np_{m}\frac{e^{r_{s}t}-1}{r_{s}}]\}. \end{array}$$

## Appendix F: Distribution of recurrence time

The probability distribution of the time that a lineage, consisting initially of a single cell, will reach size *N* as time *t* is given by the Gumbel distribution ${\text{Gumb}}_{\text{max}}(\frac{\log\;Np_{m}}{r_{m}},\frac{1}{r_{m}})$ (Avanzini and Antal 2019) with probability density function:

$$G\left(t\right)=e^{-p_{m}Ne^{-r_{m}t}}.$$

A mutant lineage initiated at time *s*, through aneuploidy, at rate $v\lambda_{a}p_{m}a_{s}$ reaches size *N* before time *t* with probability G(t−s), where s≤t. As a result, the number of successful mutant lineages which reach size *N* by time *t* can be approximated by inhomogeneous Poisson random variable with rate:

$$r\left(t\right)=v\lambda_{a}p_{m}\int_{0}^{t}a_{s}G\left(t-s\right)\;ds,$$

where $a_{s}$ is aneuploid population size at time *s* defined in equation (C4). The proliferation time is defined as the first time the size of all lineages reaches *N*. When $N\ll |r_{s}|\;|r_{a}|/uv\lambda_{s}\lambda_{a}p_{m}$ there is at most a single mutant lineage that will survive and reach size *N* (Supplementary Fig. 3) and the probability that the size of that lineage has not reached *N* by time *t* is given by:

(F1 )
$$\begin{array}{rl} P\left(m_{t}\leq N\right) & =\exp\;\left[-r\left(t\right)\right]\\ & =\exp\;\left[-\frac{Nuv\lambda_{s}\lambda_{a}p_{m}}{r_{s}-r_{a}}\int_{0}^{t}\left[e^{r_{s}x}-e^{r_{a}x}\right]e^{-p_{m}Ne^{-r_{m}\left(t-x\right)}}\;dx\right]. \end{array}$$

When $N\gg |r_{s}|\;|r_{a}|/uv\lambda_{s}\lambda_{a}p_{m}$ the dynamics of the cancer cell populations is deterministic and approximated by the system of ODEs shown in equation (D2). As a result, the size of the mutant cell population will always be below *N* until time $\tau_{a}^{r}$ and will always be greater after:

(F2 )
$$P\left(m_{t}\leq N\right)=1-H\left(t-\tau_{a}^{r}\right),$$

where H(x) is the Heaviside function:

$$H\left(x\right)=\left\{ \begin{array}{cc} 0, & x<0,\\ 1, & x\geq 0. \end{array}\right.$$

We plot equations (F1) and (F2) in Fig. 6b and compare with stochastic simulations and observe that our approximations are in agreement.

We observe that for $N=10^{7}$ our formula overestimates the probability that the mutant population will be smaller than *N* at time *t*. This can be explained by the fact that $N=10^{7}$ is an intermediary scenario where the sensitive population produces a number of rescue lineages that is greater than one but still sufficiently small such that stochasticity plays an important role in the population dynamics. As a result, the number of mutant cancer cells will reach *N* faster than the scenario with a single mutant lineage. Additionally, we observe from Fig. 6b that the probability of the mutant cell population reaching size *N* is approximately zero before time $\tau_{a}^{r}$ which is the recurrence time for the deterministic scenario. This can be explained as follows: in the deterministic scenario there is a sufficient number of lineages produced such that there exists a lineage where each descendant will only reproduce and not die; the time it takes for this lineage to reach *N* is the lower bound for the time of all other lineages to reach *N* and this time cannot be smaller than $\tau_{a}^{r}$ by definition. Given that for small values of *N* we expect that at most a single lineage will rescue the tumor, this lineage cannot reach *N* before $\tau_{a}^{r}$ for the deterministic scenario equation (D4).

From equation (F1), we obtain the distribution of the recurrence time conditional of evolutionary rescue:

(F3 )
$$f\left(t\right)=\frac{d}{dt}\left[\frac{P\left(m_{t}\geq N\right)}{p_{\mathrm{rescue}}}\right]=r^{\prime }\left(t\right)\frac{\exp\;\left[-r\left(t\right)\right]}{p_{\mathrm{rescue}}},$$

which we plot in Supplementary Fig. 4 and compare with simulations. We note that in the scenario $N\gg |r_{s}|\;|r_{a}|/uv\lambda_{s}\lambda_{a}p_{m}$ the distribution becomes the Dirac *δ*-function (Barton 1989).

## Appendix G: Bootstrap

For the mean times, the 95% confidence interval is obtained through bootstrapping in the following steps: (1) we simulate *T* 100 times; (2) we sample with replacement which we store in $T^{\prime }$; (3) for each element of this sample we obtain $\tau =E[T^{\prime }]$; (4) we repeat steps (2)–(3) 100 times to obtain *τ* and we select the upper and lower limits such that 95% of the values of *τ* lie in the interval given by the bounds.

For the threshold tumor sizes, the 95% confidence interval is obtained through bootstrapping in the following steps: (1) we simulate $p_{\mathrm{rescue}}$ 100 times; (2) we sample with replacement which we store in *S*; (3) for each element of this sample we obtain $N_{a}^{*}=1/p_{s}$ using $p_{s}=-1/N_{e}\log\;(1-\bar{S})$ where $\bar{S}$ is the mean of *S* and $N_{e}$ is an arbitrary value of the initial population size we selected in order to calculate $p_{\mathrm{rescue}}$; (4) we repeat steps (2)–(3) 100 times to obtain $N_{a}^{*}$ and we select the upper and lower limits such that 95% of the values of $N_{a}^{*}$ lie in the interval given by the bounds.

For the ratio of the threshold tumor sizes, the 95% confidence interval is obtained through bootstrapping in the following steps: (1) we simulate $p_{\mathrm{rescue}}$ 100 times for both the scenario when $f=\tilde{u}\lambda_{s}/c$ and f=0; (2) we sample with replacement which we store in $S_{f}$ and $S_{0}$; (4) for each element of $S_{0}$ we obtain $N_{a}^{*}=1/p_{s}$ using $p_{s}=-1/N_{e}\log\;(1-\bar{S})$ where $\bar{S}$ is the mean of $S_{0}$ and $N_{e}$ is an arbitrary value of the initial population size we selected in order to calculate $p_{\mathrm{rescue}}$; (5) for each element of $S_{f}$ we obtain ${\tilde{N}}_{a}^{*}=1/p_{a}$ using $p_{a}=-f/N_{e}\log\;(1-\bar{S})$ where ${\bar{S}}_{f}$ is the mean of $S_{f}$ and $N_{e}$ is an arbitrary value of the initial population size we selected in order to calculate $p_{\mathrm{rescue}}$; (6) we repeat steps (2)–(5) 100 times to obtain ${\tilde{N}}_{a}^{*}/N_{a}^{*}$ and we select the upper and lower limits such that 95% of the values of ${\tilde{N}}_{a}^{*}/N_{a}^{*}$ lie in the interval given by the bounds.

## Appendix H: Aneuploidy-induced mutation rate

The mutation rate may be increased in aneuploid cells. To account for an increased mutation rate in cells with the aneuploidy that provides a fitness advantage in the presence of a drug, we extend our model such that sensitive cells mutate with rate $v_{s}$ and aneuploid cells mutate with rate $v_{a}$. Note that sensitive cells include those cells with any other aneuploidy, including those that may cause an increased mutation rate, and therefore those cases are already covered by the model presented in the main text. We then calculate the survival probabilities $p_{s}$, $p_{a}$, and $p_{s}$ as in Appendix A,

(H1 )
$$\begin{array}{rl} 1-p_{s} & =\frac{\mu_{s}}{\lambda_{s}+\mu_{s}+u\lambda_{s}+v_{s}\lambda_{s}}\\ & \;+\frac{u\lambda_{s}}{\lambda_{s}+\mu_{s}+u\lambda_{s}+v_{s}\lambda_{s}}\left(1-p_{a}\right)\left(1-p_{s}\right)\\ & \;+\frac{\lambda_{s}}{\lambda_{s}+\mu_{s}+u\lambda_{s}+v_{s}\lambda_{s}}{\left(1-p_{s}\right)}^{2}\\ & \;+\frac{v_{s}\lambda_{s}}{\lambda_{s}+\mu_{s}+u\lambda_{s}+v_{s}\lambda_{s}}\left(1-p_{m}\right)\left(1-p_{s}\right),\\ 1-p_{a} & =\frac{\mu_{a}}{\lambda_{a}+\mu_{a}+v_{a}\lambda_{a}}\\ & \;+\frac{v_{a}\lambda_{a}}{\lambda_{a}+\mu_{a}+v_{a}\lambda_{a}}\left(1-p_{m}\right)\left(1-p_{a}\right)\\ & \;+\frac{\lambda_{a}}{\lambda_{a}+\mu_{a}+v_{a}\lambda_{a}}{\left(1-p_{a}\right)}^{2},\\ 1-p_{m} & =\frac{\mu_{m}}{\lambda_{m}+\mu_{m}}+\frac{\lambda_{m}}{\lambda_{m}+\mu_{m}}{\left(1-p_{m}\right)}^{2}. \end{array}$$

Solving the above equations, we obtain

(H2 )
$$\begin{array}{rl} \;p_{s} & =\frac{\lambda_{s}-\mu_{s}-u\lambda_{s}p_{a}-v_{s}\lambda_{s}p_{m}}{2\lambda_{s}}\\ & \;+\frac{\sqrt{{\left(\lambda_{s}-\mu_{s}-u\lambda_{s}p_{a}-v_{s}\lambda_{s}p_{m}\right)}^{2}+4\lambda_{s}^{2}\left(up_{a}+v_{s}p_{m}\right)}}{2\lambda_{s}},\\ p_{a} & =\frac{\lambda_{a}-\mu_{a}-v_{a}\lambda_{a}p_{m}+\sqrt{{\left(\lambda_{a}-\mu_{a}-v_{a}\lambda_{a}p_{m}\right)}^{2}+4\lambda_{a}^{2}v_{a}p_{m}}}{2\lambda_{a}},\\ p_{m} & =\frac{\lambda_{m}-\mu_{m}}{\lambda_{m}}. \end{array}$$

The threshold population size can be written as

(H3 )
$$N_{a}^{*}\approx \frac{\left|r_{s}\right|}{u\lambda_{s}}\cdot \left\{ \begin{array}{cc} \frac{\left|r_{a}\right|}{v_{a}\lambda_{a}}\frac{\lambda_{m}}{r_{m}},\; & r_{a}T^{*}\ll -1\;(\text{tolerant aneuploids}),\\ 2\lambda_{a}T^{*}, & -1\ll r_{a}T^{*}\ll 1\;(\text{stationary aneuploids}),\\ \frac{\lambda_{a}}{r_{a}},\; & r_{a}T^{*}\gg 1\;(\text{resistant aneuploids}), \end{array}\right.$$

where $T^{*}=(4v_{a}\lambda_{a}^{2}p_{m}{)}^{-\frac{1}{2}}$. This is the same as in equation (3) except with $v_{a}$ instead of *v*.

The probability of evolutionary rescue is

(H4 )
$$p_{\mathrm{rescue}}=1-{\left(1-p_{s}\right)}^{N}\approx 1-e^{-Np_{s}}=1-e^{-N/N_{a}^{*}},$$

which we plot in Supplementary Fig. 10 for multiple values of $v_{a}$. We note that when $v_{a}=10^{-5}$, we are in the case of stationary aneuploidy (i.e. $r_{a}T^{*}\approx -0.55$ ).
