# Supplementary material for: Evolutionary rescue by aneuploidy in tumors exposed to anticancer drugs
Source: Genetics. 2025 May 22;230(3):iyaf098. doi: 10.1093/genetics/iyaf098 (PMC12239212; doi:10.1093/genetics/iyaf098)
Supplement: iyaf098_Supplementary_Data [file iyaf098_supplementary_data.pdf]

Supplementary Figures to: Evolutionary rescue by aneuploidy in tumors exposed to anti-cancer drugs

Remus Stana[1], Uri Ben-David[2], Daniel B. Weissman[3], and Yoav Ram[1,*]

[1]School of Zoology, Faculty of Life Sciences, Tel Aviv University, Tel Aviv, Israel
[2]Department of Human Molecular Genetics and Biochemistry, Faculty of Medicine, Tel Aviv University, Tel Aviv, Israel
[3]Department of Physics, Emory University, Atlanta, GA, USA
[*]Corresponding author: Yoav Ram (e-mail: yoavram@tauex.tau.ac.il)

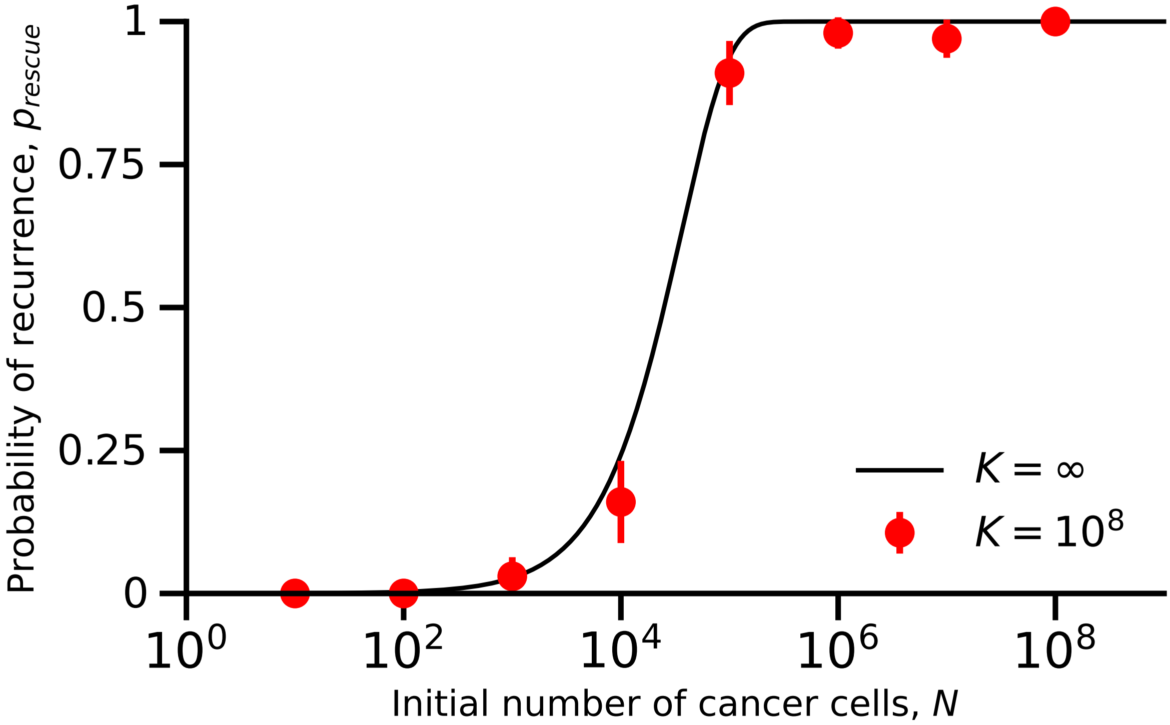

Figure S1: Density dependent growth does not affect the accuracy of our model. Comparison of results of simulations with density-dependent growth (red markers with with 95% CI) and the approximation formula (black line, eq. (3) in eq. (1)) with maximum carrying capacity $K = 10^8$ and effective carrying capacity $K_e = Kr_a/\lambda_a \approx 10^6$. The error bars represent 95% confidence interval of the form $p \pm 1.96\sqrt{p\left(1-p\right)/n}$ where p is the fraction of simulations in which the tumor has adapted to the stress and $n = 100$ is the number of simulations. Parameters: $\lambda_s = 0.1, \lambda_a = 0.0901, \lambda_m = 0.1, \mu_s = 0.14, \mu_a = 0.09, \mu_m = 0.09, u = 10^{-2}, v = 10^{-7}, K = 10^8$.

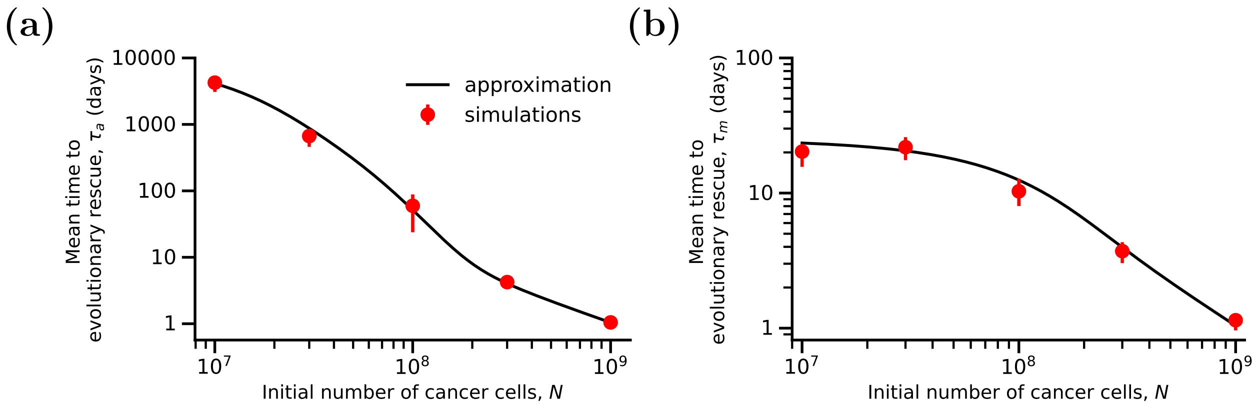

Figure S2: Evolutionary rescue time. Shown is the mean time for appearance of a resistance mutation that leads to evolutionary rescue (a) with aneuploidy ($u > 0$) and (b) without aneuploidy ($u = 0$). Our inhomogeneous Poisson-process approximations (solid black lines, right: eq. (C2), left: eq. (C7)) are in agreement with simulation results (red markers with 95% quantile intervals obtained with bootstrapping, see Appendix G). Parameters: $\lambda_s = 0.1, \lambda_a = 0.0899, \lambda_m = 0.1, \mu_s = 0.14, \mu_a = 0.09, \mu_m = 0.09, u = 10^{-2}, v = 10^{-7}$.

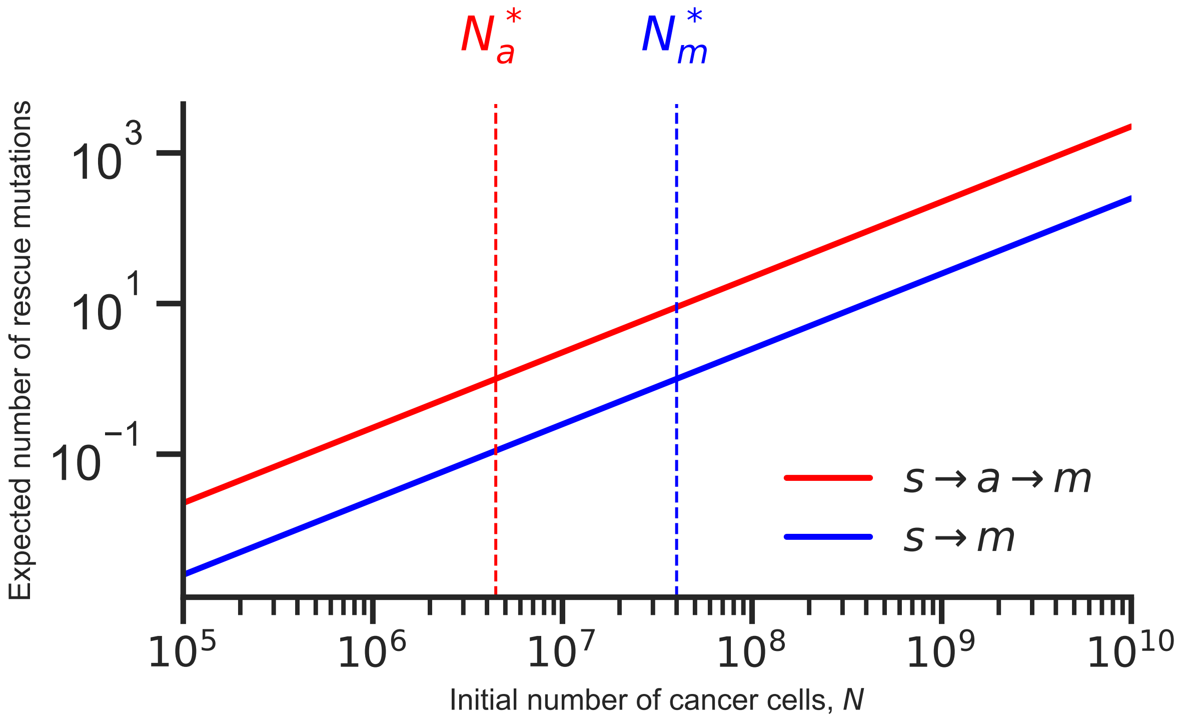

Figure S3: Aneuploidy increases the number of rescue mutations. Shown is the expected number of mutations, which will rescue the cancer cell population, produced through the evolutionary trajectory *sensitive* $\longrightarrow$ *mutant* (blue line, eq. (C6)) or through the trajectory *sensitive* $\longrightarrow$ *aneuploid* $\longrightarrow$ *mutant* (red line, eq. (C5)). Dashed vertical red line represents the threshold tumor size above which evolutionary rescue is likely through aneuploidy eq. (3) and the dashed vertical blue line represents the threshold tumor size above which evolutionary rescue is likely to occur through direct mutation eq. (2). Parameters: $\lambda_s = 0.1, \lambda_a = 0.0899, \lambda_m = 0.1, \mu_s = 0.14, \mu_a = 0.09, \mu_m = 0.09, u = 10^{-2}, v = 10^{-7}$.

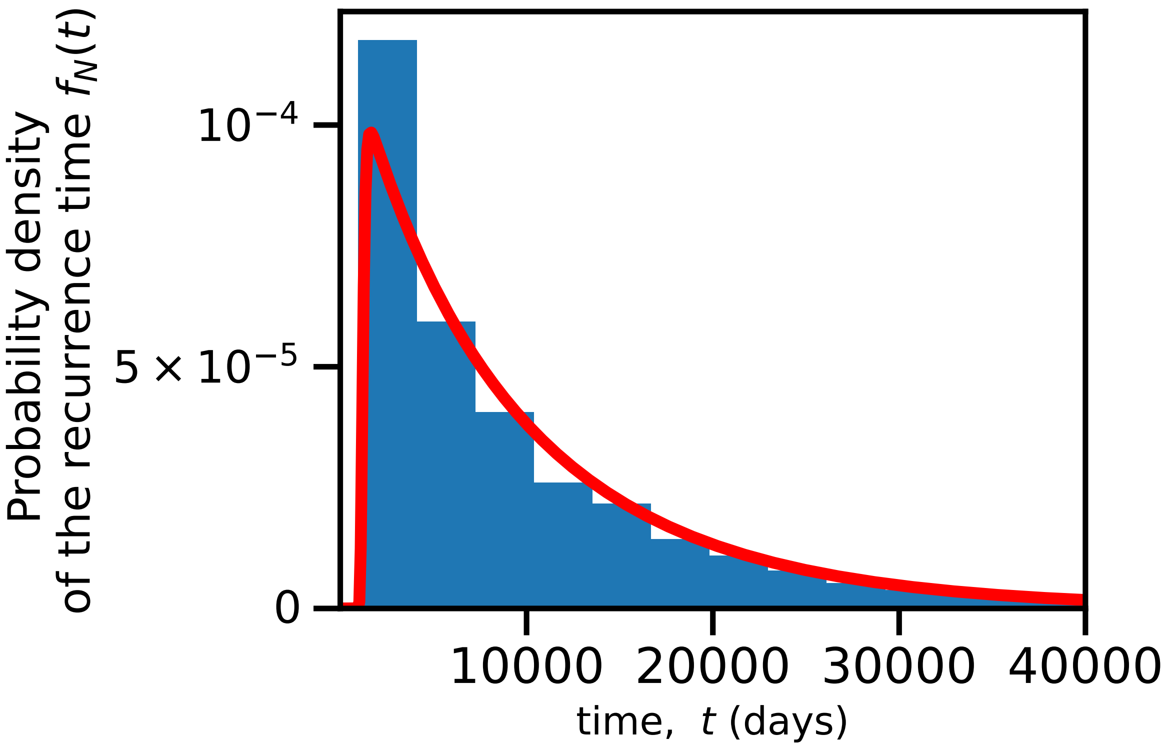

Figure S4: Distribution of the recurrence time. Shown is the distribution of the time for the mutant cell population to reach size N, where N is the initial number of cancer cells. The red line is analytic result eq. (F3) overlaid over the histogram of simulations. Parameters: $N = 10^6, \lambda_s = 0.1, \lambda_a = 0.0899, \lambda_m = 0.1, \mu_s = 0.14, \mu_a = 0.09, \mu_m = 0.09, u = 10^{-2}, v = 10^{-7}$.

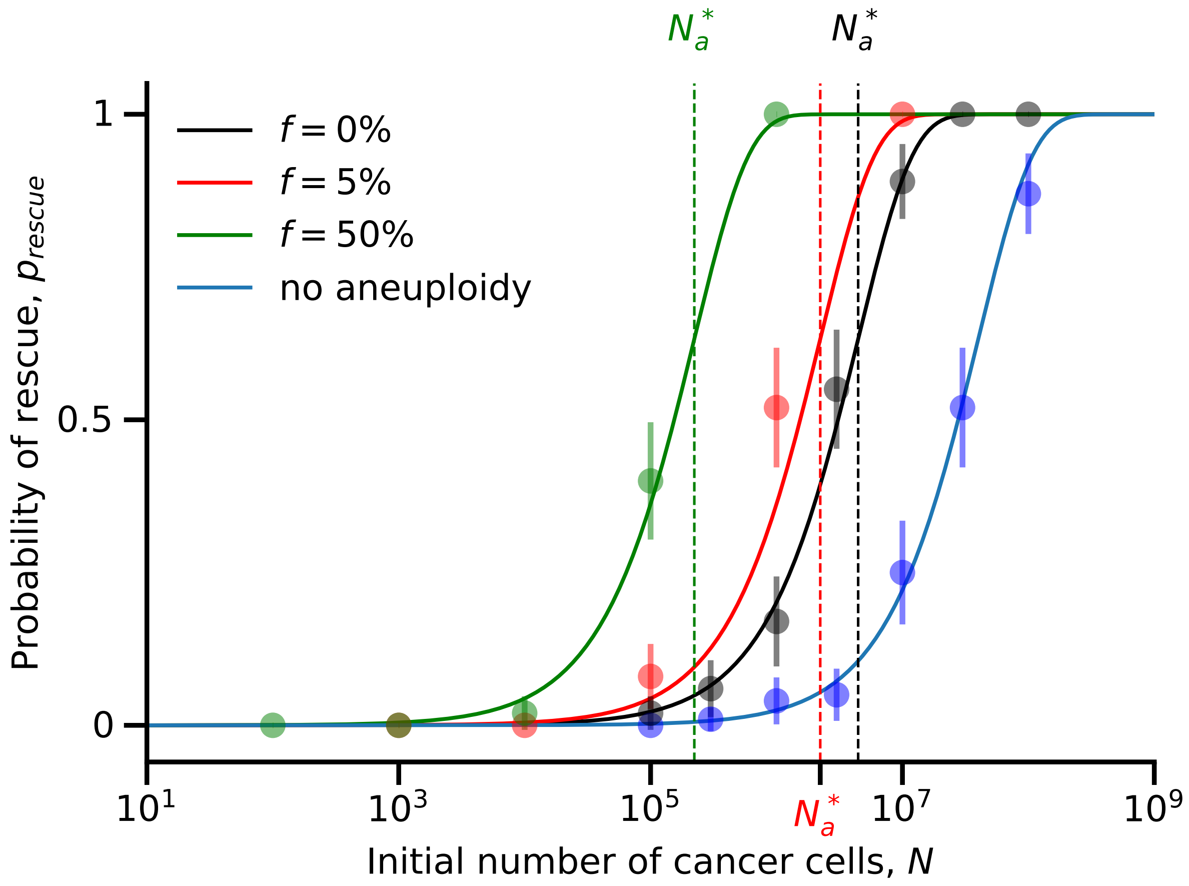

Figure S5: Standing genetic variation reduces tumor threshold size. The probability of evolutionary rescue (i.e., the probability that the population does not go to extinction), p_{rescue}, as a function of the initial tumor size, N. Dashed vertical line shows the threshold tumor size, above which the probability is high. Blue dashed line represents the probability of evolutionary rescue as a function of N without aneuploidy ($u = 0$). The black line represents the scenario where a fraction $f = 0\%$ of the initial tumor is aneuploid, the red line represents the scenario with $f = 5\%$ and the green line represents the scenario with $f = 50\%$. The dots represent simulation results and the error bars represent 95% confidence intervals ($p \pm 1.96\sqrt{p\left(1-p\right)/n}$ where p is the fraction of simulations in which the tumor has adapted to the stress and $n = 100$ is the number of simulations). Parameters: $\lambda_s = 0.1, \lambda_a = 0.0899, \lambda_m = 0.1, \mu_s = 0.14, \mu_a = 0.09, \mu_m = 0.09, u = 10^{-2}, v = 10^{-7}$.

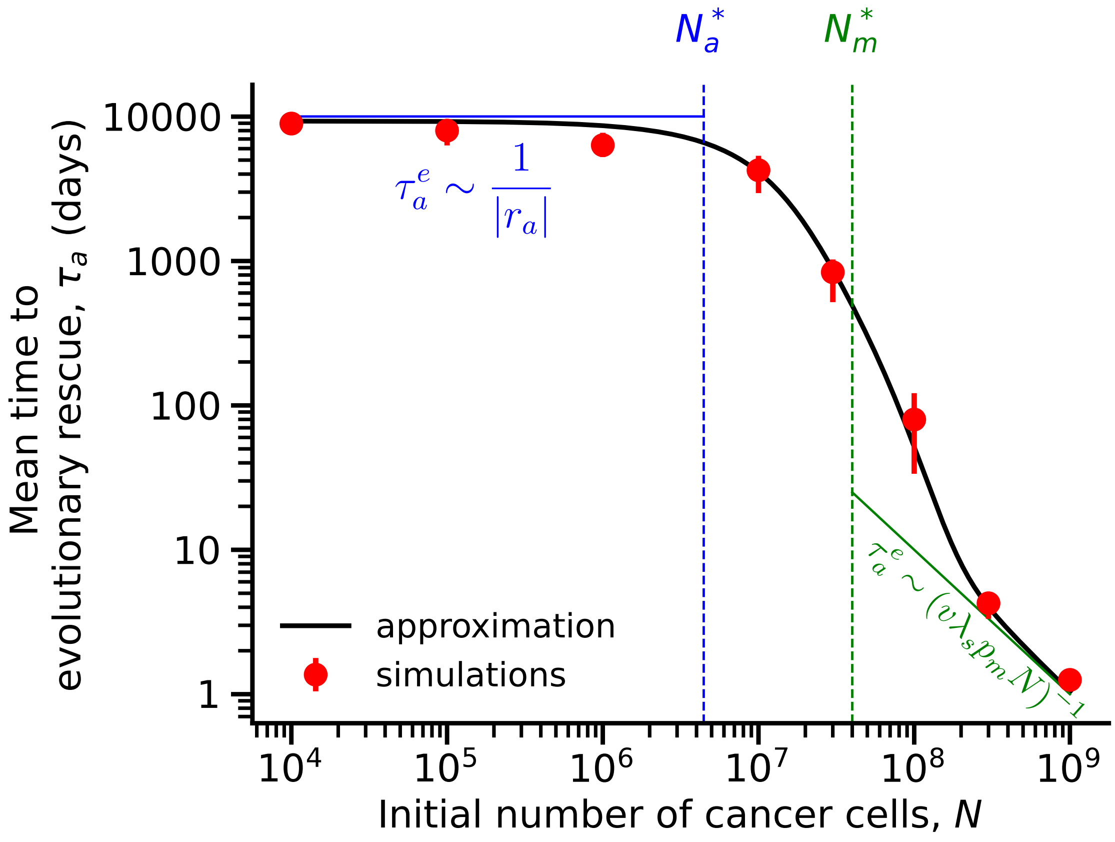

Figure S6: Tumor size reduces rescue time in a non-linear fashion. The mean time for appearance of a resistance mutation that leads to evolutionary rescue with aneuploidy ($u > 0$). Solid lines show our approximations (green: eq. (C8), blue: eq. (C9), black: eq. (C7)) compared to red markers that show mean of simulations results (with error bars for 95% confidence intervalues obtained with bootstrap, see Appendix G). Blue dashed line, N_a^*. Green dashed line, N_m^*. Parameters: $\lambda_s = 0.1, \lambda_m = 0.0899, \lambda_a = 0.1, \mu_s = 0.14, \mu_a = 0.09, \mu_m = 0.09, u = 10^{-2}, v = 10^{-7}$.

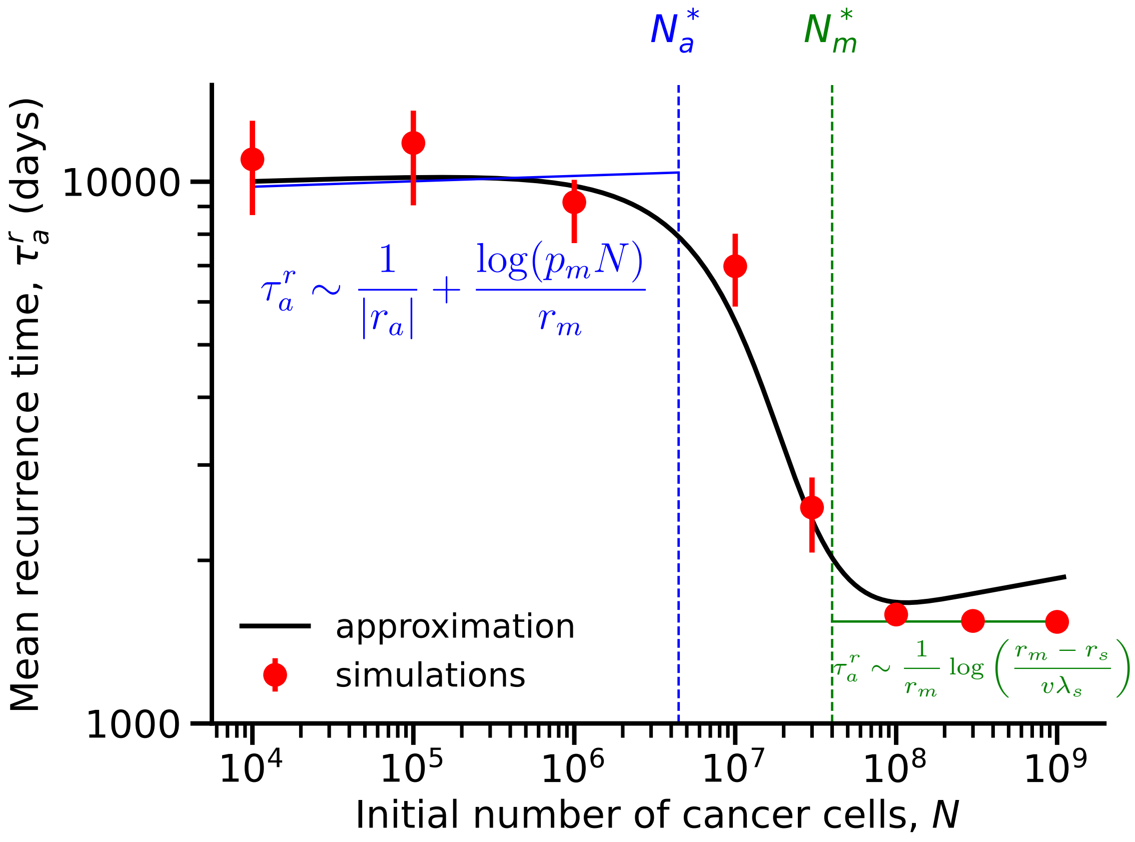

Figure S7: Tumor size reduces resucrrence time in a non-linear fashion. The mean time for the mutant cell population to reach size N, where N is the initial number of cancer cells. Solid lines show our approximations (green: eq. (D4), blue: eq. (D1) with eq. (C9) for τ_a; black: eq. (D1) with eq. (C7) for τ_a) compared to red markers that show mean of simulations results (with error bars for 95% confidence intervals obtained with bootstrap, see Appendix G). Blue dashed line, N_a^*. Green dashed line, N_m^*. Parameters: $\lambda_s = 0.1, \lambda_a = 0.0899, \lambda_m = 0.1, \mu_s = 0.14, \mu_a = 0.09, \mu_m = 0.09, u = 10^{-2}, v = 10^{-7}$.

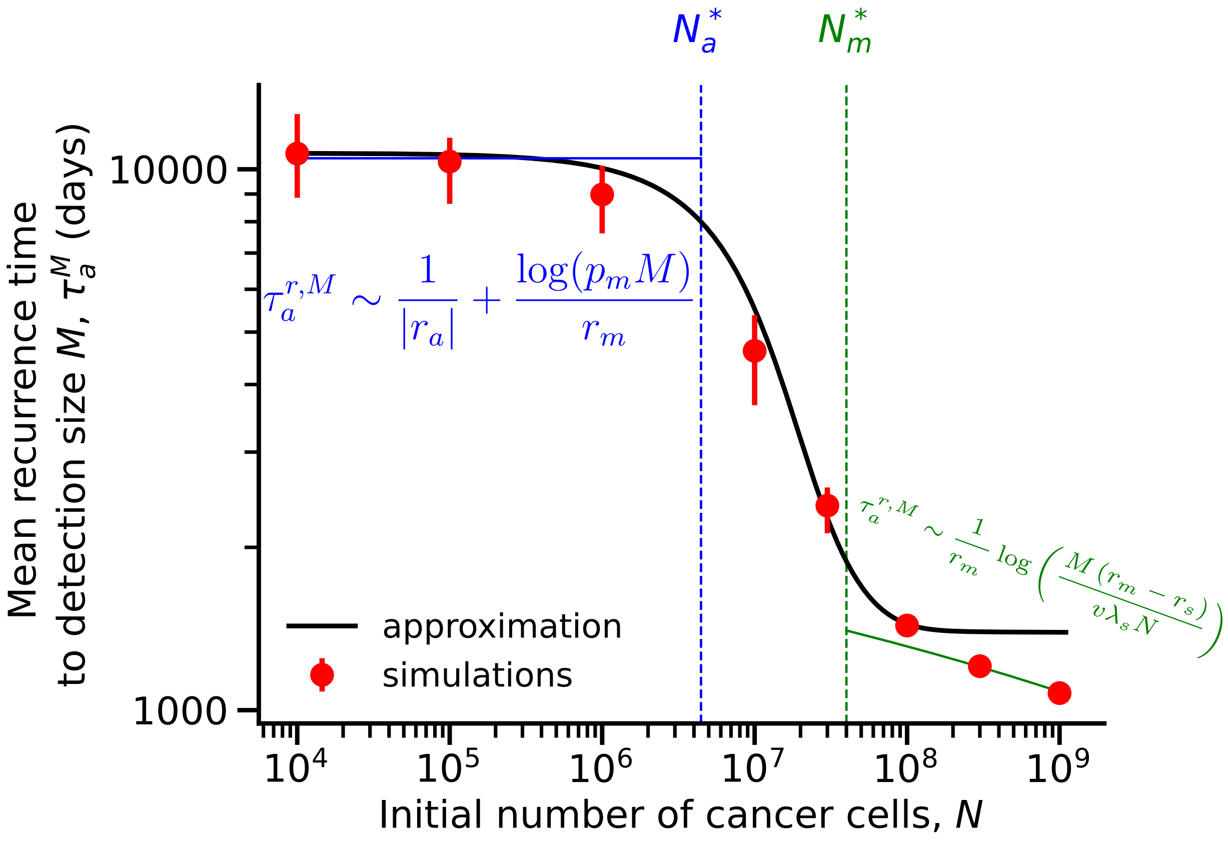

Figure S8: Tumor size reduces time to detection in a non-linear fashion. The mean time for the mutant cell population to reach size M, where M is the tumor detection size. Solid lines show our approximations (green: eq. (D6) for $N > N_m^*$, blue: eq. (D5) with τ_a from eq. (7) for $N < N_a^*$; black: eq. (D5) with τ_a from eq. (C7)) compared to red markers that show mean of simulations results (with error bars for 95% confidence intervals obtained with bootstrap, see Appendix G). Blue dashed line, N_a^*. Green dashed line, N_m^*. Parameters: $\lambda_s = 0.1$, $\lambda_a = 0.0899$, $\lambda_m = 0.1$, $\mu_s = 0.14$, $\mu_a = 0.09$, $\mu_m = 0.09$, $u = 10^{-2}$, $v = 10^{-7}$, $M = 10^7$.

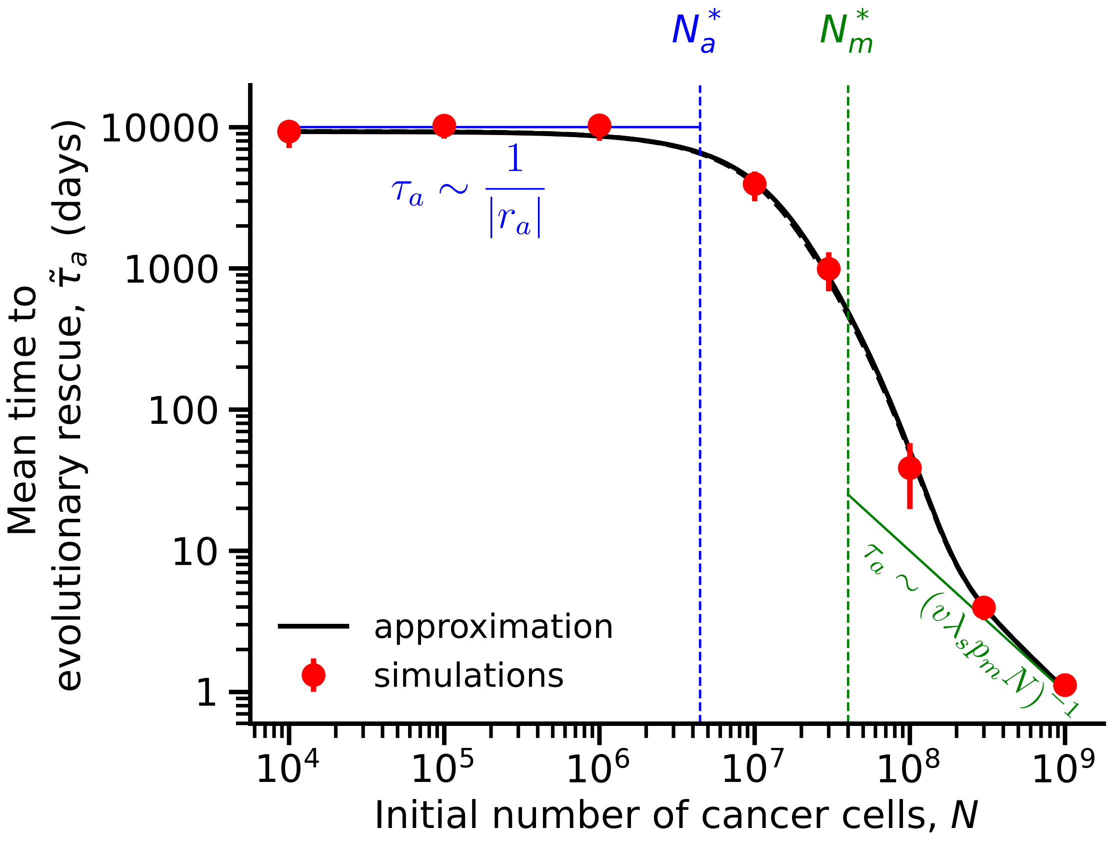

Figure S9: Tumor size reduces rescue time in a non-linear fashion: with standing genetic variation. The mean time for appearance of a resistance mutation that leads to evolutionary rescue with aneuploidy ($u > 0$) when a fraction f of cancer cells are aneuploid at the start of drug treatment. Lines show our approximations (solid green: eq. (7) for $N > N_m^*$; solid blue: eq. (7) for $N < N_a^*$; solid black: eq. (C7); dashed black: eq. (C10)) compared to red markers that show mean of simulations results (with error bars for 95% confidence intervals obtained with bootstrap, see Appendix G). Blue dashed line, N_a^*. Green dashed line, N_m^*. Parameters: $\lambda_s = 0.1, \lambda_a = 0.0899, \lambda_m = 0.1, \mu_s = 0.14, \mu_a = 0.09, \mu_m = 0.09, u = 10^{-2}, v = 10^{-7}, f = 0.14\%$.

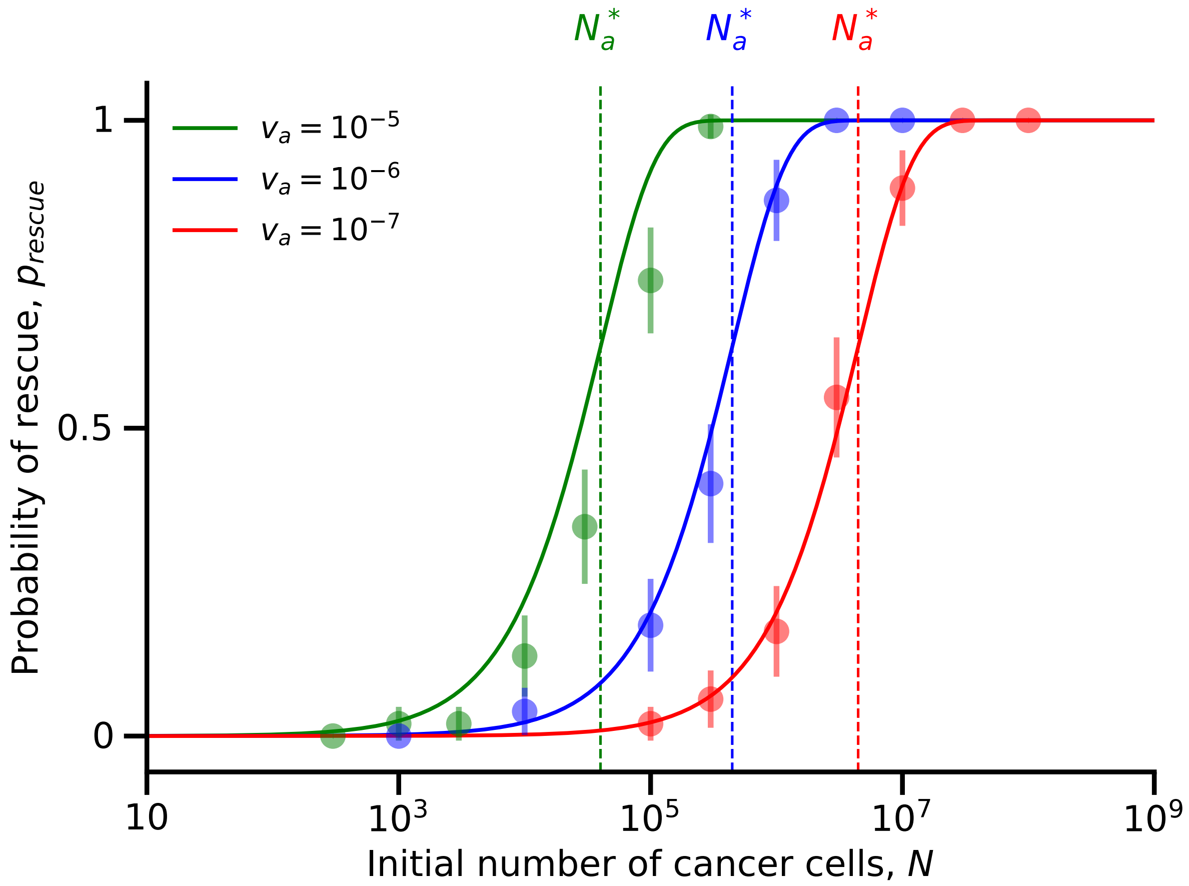

Figure S10: Effect of tumor size on probability of evolutionary rescue is sensitive to the mutation rate in aneuploid cells. The probability of evolutionary rescue (i.e., the probability that the population does not become extinct), p_{rescue}, as a function of the initial tumor size, N (eq. (1)). Dashed vertical line shows the threshold tumor size, N_a^*, above which the probability is high (eq. (H3)). Red dashed line: low mutation rate in aneuploid cells, $v_a = 10^{-7}$. Blue line: interemediate mutation rate in aneuploid cellsm $v_a = 10^{-6}$. Green line: high mutation rate in aneuploid cells, $v_a = 10^{-5}$. Markers for simulation results, with error bars for 95% confidence intervals ($p \pm 1.96\sqrt{p\left(1-p\right)/n}$ where p is the fraction of simulations in which the tumor has been rescued and $n = 100$ is the number of simulations). Parameters: $\lambda_s = 0.1, \lambda_m = 0.1, \mu_s = 0.14, \mu_a = 0.09, \mu_m = 0.09, u = 10^{-2}, v_s = 10^{-7}$.
